# Region-specific Wnt signaling responses promote gastric polyp formation in patients with familial adenomatous polyposis

**DOI:** 10.1172/jci.insight.174546

**Published:** 2023-11-09

**Authors:** Kevin P. McGowan, Elizabeth Delgado, Theresa M. Keeley, Elise S. Hibdon, D. Kim Turgeon, Elena M. Stoffel, Linda C. Samuelson

**Affiliations:** 1 Department of Molecular & Integrative Physiology and; 2 Department of Internal Medicine, University of Michigan Medical School, Ann Arbor, Michigan, USA.

**Keywords:** Gastroenterology, Adult stem cells, Gastric cancer, Tumor suppressors

## Abstract

Germline adenomatous polyposis coli (*APC*) mutation in patients with familial adenomatous polyposis (FAP) promotes gastrointestinal polyposis, including the formation of frequent gastric fundic gland polyps (FGPs). In this study, we investigated how dysregulated Wnt signaling promotes FGPs and why they localize to the corpus region of the stomach. We developed a biobank of FGP and surrounding nonpolyp corpus biopsies and organoids from patients with FAP for comparative studies. Polyp biopsies and polyp-derived organoids exhibited enhanced Wnt target gene expression. Polyp-derived organoids with intrinsically upregulated Wnt signaling showed poor tolerance to further induction, suggesting that high Wnt restricts growth. Targeted genomic sequencing revealed that most gastric polyps did not arise via *APC* loss of heterozygosity. Studies in genetic mouse models demonstrated that heterozygous *Apc* loss increased epithelial cell proliferation in the corpus but not the antrum, while homozygous *Apc* loss was not maintained in the corpus yet induced hyperproliferation in the antrum. Our findings suggest that heterozygous *APC* mutation in patients with FAP may be sufficient to drive polyp formation in the corpus region while subsequent loss of heterozygosity to further enhance Wnt signaling is not tolerated. This finding contextualizes the abundant yet benign nature of gastric polyps in FAP patient corpus compared with the rare, yet adenomatous polyps in the antrum.

## Graphical abstract

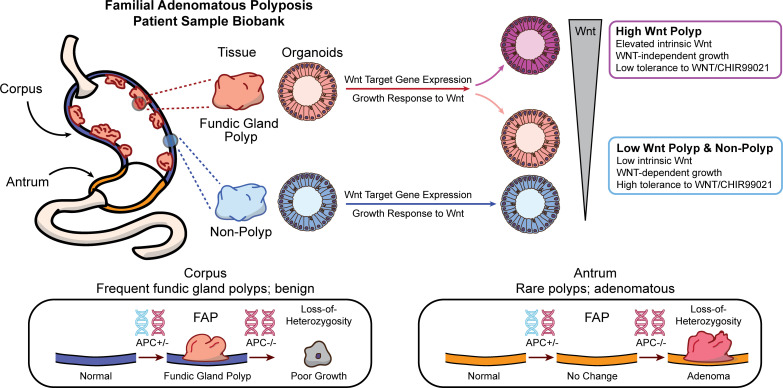

## Introduction

Familial adenomatous polyposis (FAP) is an autosomal dominant disease resulting from an inherited loss-of-function mutation to the tumor suppressor adenomatous polyposis coli (*APC*). *APC* mutation leads to activation of the Wnt signaling pathway, and thus patients with FAP are predisposed to developing disease in numerous tissues (1, 2). The most significant disease burden occurs within the gastrointestinal tract, where, in the absence of endoscopic or surgical intervention, patients with FAP have a more than 90% effective risk of developing colorectal cancer throughout their lifetime (2–4).

Patients with FAP are also at an increased risk for polyposis within the stomach. The abundance of gastric polyps within patients with FAP varies, with some patients developing hundreds to thousands of lesions and often leading to carpeting of the gastric corpus (5). These polyps predominately arise as fundic gland polyps (FGPs), which are sessile lesions characterized by cystically dilated glands (6–9). FGPs in patients with FAP sometimes present with features of low-grade dysplasia, but rarely demonstrate high-grade dysplasia, and are typically considered benign (5, 10–12). These polyps are also regionally restricted to the corpus, with gastric polyps in patients with FAP only rarely found within the antral region. The rare antral polyps that emerge typically exhibit adenomatous change and have increased potential to progress to cancer in line with colorectal polyps (13).

The mechanisms through which Wnt signaling underscores FGP emergence remain unclear as little is known about how Wnt regulates corpus epithelial homeostasis in humans. Furthermore, it is unknown why FGPs rarely progress to cancer while colorectal cancer in these patients is so prevalent. Although *APC* mutations have been detected in 7%–34% of gastric cancer, patients with FAP are at only a 0.5%–1.3% lifetime risk of developing gastric cancer (13–17). The mechanism of polyp emergence within the colon would suggest that additional somatic hits to *APC*, including loss of heterozygosity, initiates formation; however, few studies have investigated the mutational landscape of FGPs in patients with FAP (18).

The “just-right” hypothesis describes an optimal range of Wnt signaling to drive cellular growth and proliferation in the context of polyposis. This principle has been used to describe the regional distribution of cancer throughout the colon but has not been explored within the stomach (19–23). We have recently demonstrated that corpus organoids derived from patients with no known underlying gastric disease exhibited a lower threshold for Wnt signaling to drive optimal growth relative to individual-matched antral organoids (24). This finding predicts that, at baseline, corpus stem/progenitor cells may be more susceptible to develop hyperplasia upon Wnt activation compared with antral stem cells, but this concept and the subsequent application of the “just-right” hypothesis underscoring FGP development have not been defined.

In this study, we investigated the etiology of FGP formation resulting from germline *APC* mutations in patients with FAP. We established a biobank of patient-matched FGP and surrounding nonpolyp gastric tissue samples to conduct comparative analyses of genomic DNA sequencing, mRNA expression analysis, and in vitro organoid growth experiments. We demonstrate that FGPs have increased expression of Wnt target genes relative to their patient-matched nonpolyp samples, indicating upregulation of the Wnt signaling pathway. However, additional somatic alterations to *APC* indicating loss of heterozygosity were infrequent, demonstrating that this mechanism does not underscore enhanced Wnt signaling and is not required for polyp emergence. Ultimately, we demonstrate through human FAP organoids and genetic mouse models that heterozygous loss of *APC* optimally drives corpus proliferation while homozygous loss is not tolerated.

## Results

### Enhanced Wnt target gene expression in FAP-associated FGPs.

**Figure 1:**
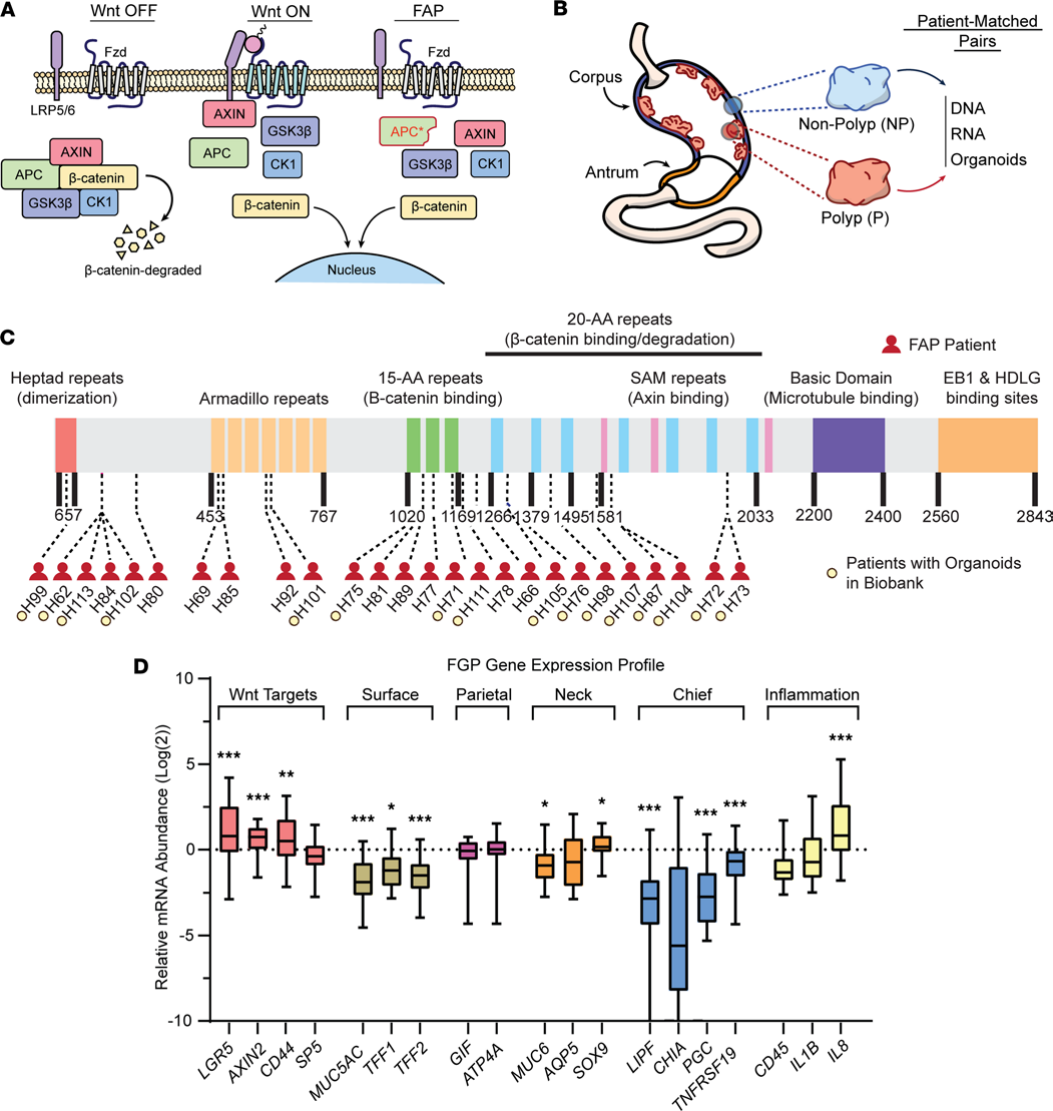
FGP biopsy samples from patients with FAP have increased Wnt target gene expression. (**A**) Schematic of Wnt signaling in Wnt OFF, in Wnt ON, and in FAP patients with mutated APC (APC*) exhibiting nuclear localization of β-catenin in the absence of Wnt ligand. (**B**) Biopsies from paired fundic gland polyps (P) and surrounding nonpolyp (NP) corpus tissue were collected to establish an FAP patient biobank of RNA, DNA, and organoids. (**C**) Schematic of the APC protein labeled with specific familial mutation sites for the patients in our biobank. Patients with established polyp and nonpolyp organoids are designated. Only patients with known germline mutations are included on this schematic (see Table 1). (**D**) Relative mRNA abundance of select genes in FAP patient biopsies. qPCR analysis of Wnt target–, cell marker–, and inflammation-related transcripts, with *HPRT* used as an internal reference transcript. Data are displayed as log_2_ fold-change (error bars minimum to maximum values, box length IQR, whiskers outliers) relative to patient-matched nonpolyp tissue (*n*_nonpolyp_ = 20–25 biopsies, *n*_polyp_ = 21–30 biopsies). Statistical analysis by unpaired parametric Student’s *t* test (*P < 0.05, **P < 0.005, ***P < 0.001; see Supplemental Figure 1).

*APC* mutation activates Wnt signaling through increased translocation of β-catenin to the nucleus resulting from compromised function of the destruction complex (Figure 1A). To investigate the molecular and cellular etiology of gastric FGP formation in patients with FAP, we established a biobank of patient-paired FGP (P) and surrounding nonpolyp (NP) gastric corpus tissue samples (Figure 1B). From biopsy sample pairs, we extracted genomic DNA, extracted RNA, and/or developed paired organoid cultures for further analyses. Our biobank consisted of samples from 34 individual patients with FAP across a spectrum of germline *APC* mutations and clinical features (Figure 1C, Table 1, and Supplemental Table 1; supplemental material available online with this article; https://doi.org/10.1172/jci.insight.174546DS1).

We first analyzed the gene expression profile of patient-matched P versus NP biopsies to determine whether upregulated Wnt signaling underscored FGP emergence. Quantitative PCR (qPCR) analysis of transcript abundance revealed that Wnt target genes *LGR5*, *AXIN2*, and *CD44* were upregulated in FGP biopsies relative to patient-matched NP tissue (Figure 1D and Supplemental Figure 1A). Analysis of differentiated cell markers showed that FGP biopsies were deficient in markers for surface cells, which is consistent with studies showing that upregulation of Wnt signaling alters corpus epithelial cell differentiation (Figure 1D and Supplemental Figure 1B) (24–26). FGP biopsies also showed markedly reduced expression of chief cell markers *LIPF*, *CHIA*, *PGC*, and *TNFRSF19* along with a more subdued decrease in the mucous neck cell marker *MUC6* (Figure 1D and Supplemental Figure 1, D and E). It is unknown whether these changes in cell marker expression are a consequence of biopsy extraction or due to altered stem/progenitor cell differentiation secondary to Wnt activation in FGPs. Notably, parietal cell markers *GIF* and H/K ATPase subunit *ATP4A* were unchanged (Figure 1D and Supplemental Figure 1C). Analysis of inflammatory markers showed no difference in *CD45* expression, suggesting that FGP formation was not associated with inflammatory cell influx, although some polyps exhibited a pro-inflammatory environment suggested by increased expression of *IL8* (Figure 1D and Supplemental Figure 1F).

### Wnt tone is intrinsically elevated in high-Wnt FGP-derived organoids.

**Figure 2:**
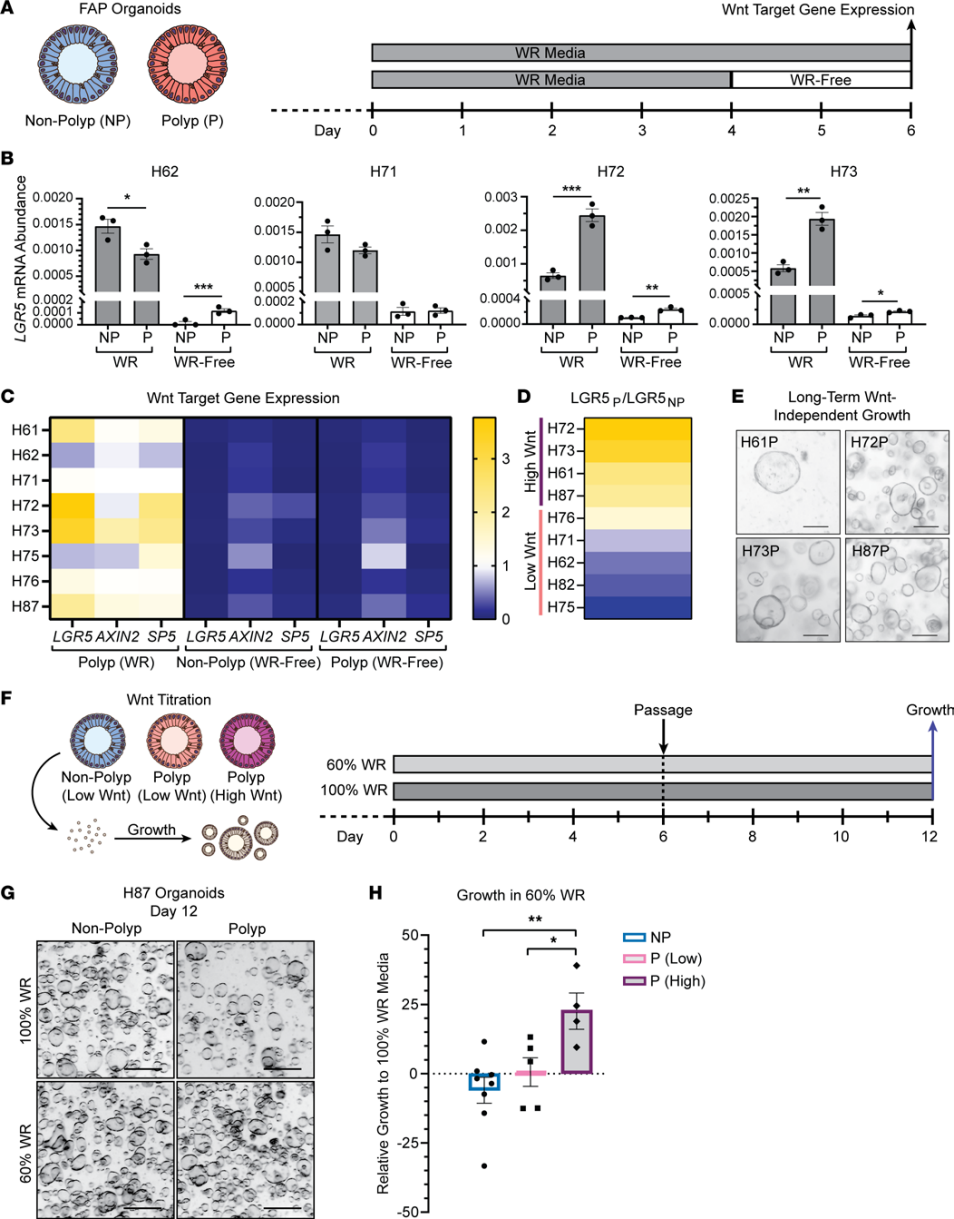
Enhanced Wnt signaling in a subset of FAP gastric polyp-derived organoids. (**A**) Wnt target gene expression was measured in patient-matched polyp (P)/nonpolyp (NP) organoid pairs following 6 days of growth in WR media or following 4 days of growth in WR and 2 days of growth in WR-free media. (**B**) *LGR5* mRNA abundance in FAP organoids grown in WR or WR-free media. Data shown as mean ± SD mRNA abundance relative to the reference gene *ACTB* (*n* = 3 individual wells; *P < 0.05, **P < 0.005, ***P < 0.001 by 1-way ANOVA with Tukey’s multiple-comparison test). (**C**) Heatmap of relative Wnt target gene expression in polyp organoids. Data are shown as mean fold-change relative to the expression of each target gene in matched nonpolyp organoids grown in WR media for 6 days. (**D**) Heatmap of *LGR5* mRNA expression in polyp organoids, shown as mean fold-change relative to patient-matched nonpolyp organoids and ordered from highest to lowest expression. High Wnt and low Wnt denote the classification of polyp-derived lines with increased or similar/decreased *LGR5* gene expression. (**E**) Images of polyp organoids grown for 2 passages (12 days) in WR-free media, demonstrating Wnt-independent growth (scale bars = 100 μm). (**F**) Growth of organoids cultured for 12 days in 100% or 60% WR media was measured through ATP-dependent luminescence. (**G**) Images of H87 organoids at day 12 following growth in 100% or 60% WR (scale bars = 200 μm). (**H**) Relative growth of nonpolyp and polyp organoids clustered by Wnt target gene expression characteristics (high Wnt and low Wnt) in 60% compared with 100% WR media. Each point represents an individual organoid line, shown as the average of triplicate wells. Data shown as mean ± SD of the organoids in that group (*n* = 4–9 individual organoid lines; *P < 0.05, **P < 0.005 by 1-way ANOVA with Tukey’s multiple-comparison test).

Given our observations that Wnt target gene expression was upregulated in FGPs, we tested whether Wnt signaling is intrinsically elevated in corpus progenitors by generating P- and NP-derived organoids. We passaged FAP patient–derived organoids at least 3 times before analysis to remove nonepithelial cells and to establish stable self-renewing cultures. Wnt target gene expression was measured in 9 patient-matched organoid pairs after growth in normal media containing exogenous Wnt signaling ligands (WNT, RSPO; WR media) and following transition into WNT/RSPO-depleted media (WR-free) (Figure 2A). Comparison of *LGR5* mRNA abundance in FAP patient–matched P- and NP-derived organoids revealed variability in intrinsic Wnt tone, with polyp organoids falling into 2 subgroups. In some patient pairs, such as H62 and H71, we noted either no difference in *LGR5* expression between P and NP organoids or even decreased *LGR5* in P organoids grown in WR media (Figure 2B). Other patients, such as H72 and H73, demonstrated enhanced *LGR5* expression in P organoids. In all lines, we observed that *LGR5* expression was drastically reduced following acute WNT/RSPO withdrawal, thus demonstrating that all lines remained sensitive to exogenous Wnt. Broadening our analysis to include *AXIN2* and *SP5* demonstrated consistent trends in Wnt target gene expression when normalized to patient-matched NP organoids grown in WR media (Figure 2C). Based on this analysis, we subgrouped the organoids into high-Wnt polyps with enhanced Wnt target gene expression relative to patient-matched NP organoids (H72, H73, H61, H87) and low-Wnt polyps with similar or reduced target gene expression (H76, H71, H62, H82, H75) (Figure 2D).

We further assessed intrinsic Wnt tone by growing organoids for 2 passages in WR-free media. Consistent with previous studies reporting that corpus organoids require both exogenous WNT and RSPO to be maintained over time, none of the NP or low-Wnt polyp lines were capable of sustained growth in Wnt-depleted media (25). In contrast, all 4 high-Wnt polyp lines grew long term in WR-free media, thus demonstrating long-term Wnt-independent growth (Figure 2E). Therefore, enhanced Wnt target gene expression and growth in Wnt-depleted media suggest an intrinsic increase in Wnt signaling in these FAP polyps.

To test whether high-Wnt polyp organoids grow in WR-free media because they secrete endogenous WNT ligand, we measured growth after treatment with the porcupine inhibitor LGK974, which suppresses Wnt ligand secretion (Supplemental Figure 2A). We observed that LGK974 treatment had no impact on growth of high-Wnt polyp lines H72P, H73P, or H87P (Supplemental Figure 2, B and C). We further observed that neither coculture of high-Wnt organoids growing in WR-free media with NP organoids, nor feeding of NP organoids with conditioned media from these high-Wnt lines, could rescue NP organoid growth in the absence of exogenous WNT/RSPO (Supplemental Figure 2, D–G). Therefore, we demonstrate through both pharmacologic inhibition and coculture experiments that high-Wnt polyp organoids are sustained long term in WR-free media through an intrinsic signaling mechanism rather than by secreting Wnt or other growth factors into their environment.

### Increased growth of high-Wnt FGP-derived organoids in reduced-Wnt media.

Wnt signaling in the gastrointestinal tract has been proposed to follow a principle known as the “just-right” hypothesis, where excessive growth leading to polyposis is driven by an optimal level of Wnt. While too little Wnt signaling induces differentiation, hypermorphic upregulation of Wnt signaling, such as through total loss of APC function, can also be prohibitive to growth. This principle has previously been demonstrated to underscore regional patterns of colorectal cancer emergence in patients with FAP where second-hit mutations to *APC* leading to further dysregulation of Wnt signaling are selected to preserve some residual APC function (20–23, 27). We were interested in whether this principle also holds true for growth of FAP gastric polyp organoids. We hypothesized that high-Wnt polyp organoids would have a reduced tolerance for extrinsic Wnt due to elevated intrinsic Wnt signaling and therefore would exhibit maximal growth at lower concentrations of exogenous Wnt than NP or low-Wnt P organoids. To test sensitivity to extrinsic Wnt, we grew organoids over 2 passages (12 days total) in either normal-WR (100%) or reduced-WR (60%) media to define the optimal Wnt environment for growth for each organoid line (Figure 2F). At day 12, organoids were imaged to assess overall appearance, and growth was measured using an ATP-dependent cell viability assay.

Using organoids from patient H87 as an example, we observed that NP organoids grew at similar densities in 100% and 60% WR media (Figure 2G). In contrast, H87 polyp organoids, which are in the high-Wnt subgroup, grew denser in 60% WR. Extending this analysis to all 9 patient lines, we observed that NP organoids exhibited similar growth in 60% WR media as they did in 100% WR (Figure 2H). While low-Wnt polyp-derived organoids had, on average, no change in growth between the 2 conditions, polyp organoids from the high-Wnt group exhibited significantly enhanced growth in 60% WR. These results align with the “just-right” hypothesis and demonstrate an interplay between intrinsic Wnt tone and extrinsic Wnt signaling for optimal growth.

### Intrapatient variability in FGP-derived organoid growth.

**Figure 3:**
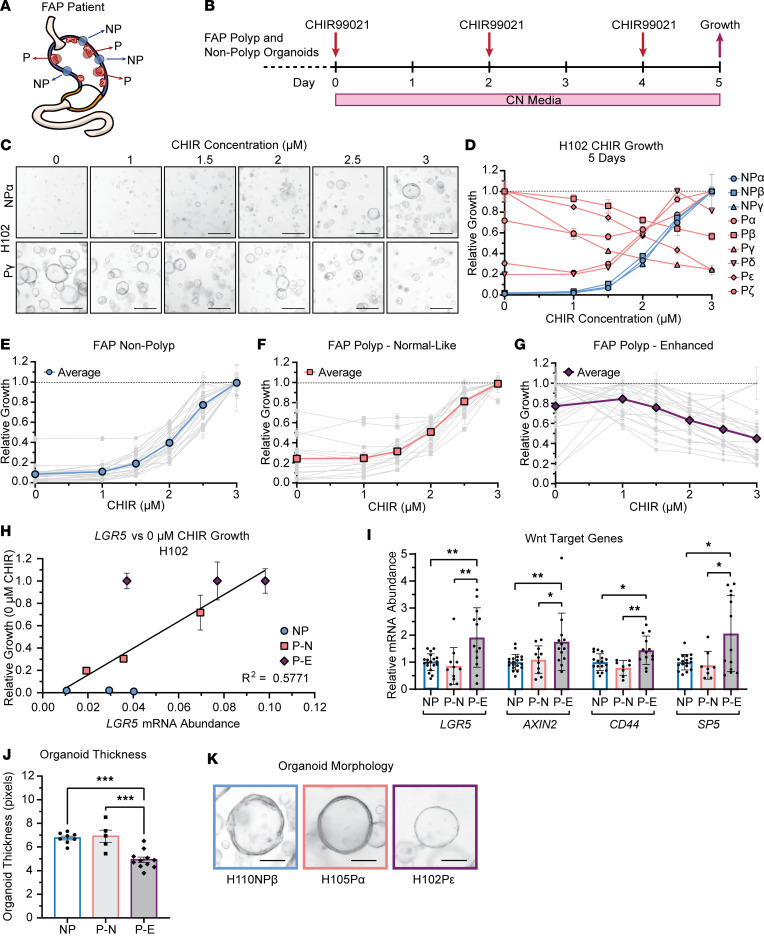
Intrinsic Wnt tone patterns intrapatient FGP variability. (**A**) Polyp (P) and nonpolyp (NP) biopsies were obtained to establish multiple polyp-derived organoids from each patient with FAP. (**B**) Organoids were grown in CN media with varying concentrations of CHIR, and growth was measured through ATP-dependent luminescence on day 5. (**C**) Representative images of H102NPα and H102Pγ following culture in 0–3 μM CHIR (scale bars = 100 μm). (**D**) Relative growth of H102 organoid lines as a function of CHIR concentration. Growth was normalized to the maximal growth observed for each line, with data shown as mean ± SD of triplicate wells. (**E**–**G**) Relative growth of organoid lines from 12 patients with FAP plotted as a function of CHIR concentration. Individual organoid lines are shown in gray, and the average of all lines is shown in color. (**E**) Nonpolyp organoid lines (blue, *n* = 23 lines). (**F**) Polyp organoid lines demonstrating normal-like growth (pink, *n* = 14 lines). (**G**) Polyp organoid lines demonstrating enhanced Wnt-independent growth (purple, *n* = 21 lines). (**H**) Relative growth of H102 organoid lines after 5 days of growth in 0 μM CHIR (WR-free media) versus *LGR5* mRNA abundance after 6 days of growth in 60% WR. The trend line was calculated via linear regression analysis. (**I**) Relative mRNA abundance of Wnt target genes in FAP organoids after 6 days’ growth in 60% WR. Data shown as mean ± SD fold-change relative to patient-matched nonpolyp. NP: *n* = 19; P-N: *n* = 11; P-E: *n* = 13 (*P < 0.05, **P < 0.005 by 1-way ANOVA with Tukey’s multiple-comparison test). (**J**) Average NP, P-N, or P-E organoid thickness after 6 days’ growth in 60% WR (***P < 0.001). (**K**) Representative images of nonpolyp, normal-like polyp, and enhanced polyp organoids after 6 days’ growth in 60% WR (scale bars = 50 μm).

We sought to further investigate the relationship between intrinsic Wnt tone and Wnt sensitivity through an analysis of additional FAP patient–derived organoids. We were also interested in whether individual polyps from the same patient would exhibit polyp-to-polyp variability or similar characteristics, which might suggest genotype-dependent mechanisms. To study this, we expanded our organoid biobank to include multiple independent P and NP samples from 17 additional patients (Figure 1C, Figure 3A, Table 1, and Supplemental Table 1). Organoids were established by directly embedding minced biopsy tissue in Matrigel, which improved establishment efficiency compared with our previous gland isolation approach. Based on our growth analysis, we established these organoids in 60% WR media to prevent selection bias or suppression of growth in lines with elevated intrinsic Wnt tone. Epithelial outgrowth was observed after 2–4 days in culture, and subsequent passaging resulted in purification of epithelial organoids (Supplemental Figure 3A). We noted that most polyp-derived biopsies developed organoids at faster rates than NP biopsies, suggesting that polyps had increased progenitor potential (Supplemental Figure 3, B and C). Organoids were passaged at least 3 times before analysis to establish pure epithelial cultures.

We evaluated Wnt sensitivity by passaging these FAP organoids into media containing the pharmacologic Wnt activator CHIR99021 (CHIR) to dose-dependently activate Wnt signaling in the absence of exogenous WNT/RSPO ligands (Figure 3B) (24). Organoids were cultured for 5 days in media containing 0–3 μM CHIR with recombinant Noggin (CN media) to assess Wnt-dependent growth.

In each patient sample set, we observed polyp-to-polyp variability in Wnt sensitivity (Figure 3 and Supplemental Figure 4). For example, from patient H102 we established organoids from 6 unique FGPs and 3 distinct NP regions. Images taken on day 5 of CN media culture showed that while NP line H102NPα had a positive growth response to increasing Wnt, polyp line H102Pγ was Wnt averse, with reduced growth in response to increased CHIR (Figure 3C). While all H102 NP lines followed the growth patterns of H102NPα, we observed that some polyp lines, including Pβ, Pγ, and Pε, showed enhanced Wnt-independent growth and reduced growth with increased Wnt signaling (Figure 3D). In contrast, increased CHIR stimulated growth in lines Pα, Pδ, and Pζ, similar to NP lines.

This CHIR growth analysis stratified the expanded patient polyp organoid pool into 2 subgroups. Regardless of patient (Table 1 and Supplemental Table 1), we found that all NP organoids followed a near-identical dose-response pattern with growth peaking at approximately 3 μM CHIR (Figure 3E). One subgroup of polyp organoids (termed normal-like or P-N) had a similar growth pattern with increased CHIR leading to increased growth (Figure 3F). The second subgroup (termed enhanced or P-E) demonstrated Wnt independence and high sensitivity to increased CHIR (Figure 3G). From 35 polyp lines, 14 (40%) were P-N and 21 (60%) were P-E. While some patients’ polyps exhibited either P-N or P-E growth characteristics, others exhibited both types (Supplemental Figure 4). This variability suggests that polyp growth characteristics are not strictly genotype determined, though the small numbers of polyps analyzed from each patient makes it difficult to reach conclusions.

To determine if P-E organoids had higher Wnt signaling tone, we cultured organoids for 6 days in 60% WR media and measured Wnt target gene expression (Figure 3, H and I, and Supplemental Figure 5A). Using H102 organoids as an example, we determined that *LGR5* expression was positively correlated with enhanced Wnt-independent growth, suggesting that upregulated Wnt tone corresponded with increased Wnt sensitivity (Figure 3H). Expanding this analysis showed that P-E organoids had significantly upregulated Wnt target gene expression relative to paired NP and P-N organoids, consistent with our previous findings and the “just-right” hypothesis (Figure 3I).

We also analyzed cell differentiation markers in these 3 organoid types (Supplemental Figure 5, B–E). We and others have shown that Wnt regulates a bimodal axis of differentiation in human and mouse corpus organoids, with low Wnt promoting surface mucous cell differentiation and high Wnt promoting neck and chief cell differentiation (24–26). Analysis of cell marker expression showed that P-E organoids had the lowest expression of surface cell markers *MUC5AC*, *TFF1*, and *TFF2*, consistent with enhanced Wnt signaling (Supplemental Figure 5B). This aligns with our previous study and verifies that intrinsic Wnt pathway activation directly suppresses surface cell differentiation similarly to exposure to exogenous WNT/RSPO ligand. Interestingly, although our biopsy analysis showed a consistent decrease in chief cell markers across all polyp samples, analysis of P-E organoids demonstrated increased expression of some (*LIPF* and *TNFRSF19*) but not all (*PGC*) chief cell markers (Supplemental Figure 5D). However, *PGC* can also localize to human gastric neck cells, and we did not observe alterations in neck cell marker expression (Supplemental Figure 5C) (28). This finding of increased chief cell marker expression in organoids with increased Wnt pathway activation is consistent with previous studies and validates the role of Wnt signaling in regulating the bimodal axis of differentiation within the corpus, with low Wnt driving surface cell differentiation and high Wnt driving chief cell differentiation (24–26).

The organoid subgroups also exhibited morphological differences. While NP and P-N polyp organoids were similar, P-E organoids were significantly thinner (Figure 3, J and K). We speculate that this may be due to the altered differentiation characteristics.

### FAP FGPs develop without requirement for APC loss of heterozygosity.

**Figure 4:**
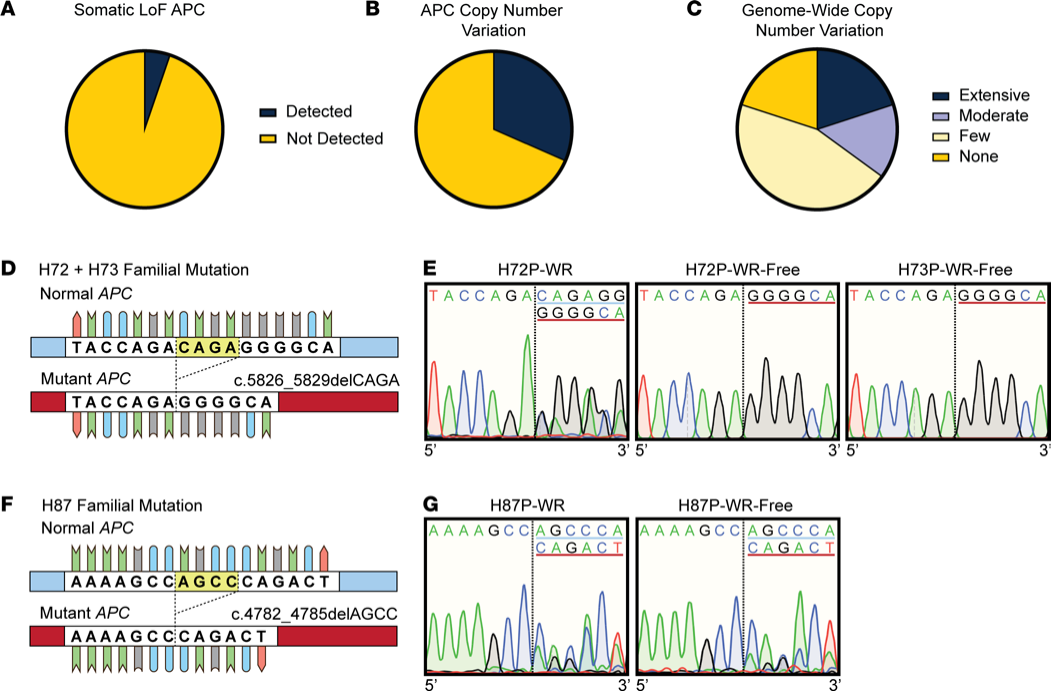
Infrequent somatic *APC* mutations in FAP patient FGPs. (**A**) Targeted sequencing of 19 FAP patient polyp and nonpolyp samples using a QIAGEN Human Comprehensive Cancer Panel detected 1 patient (H85) polyp with a novel somatic *APC* loss-of-function mutation. (**B**) A total of 6 patients, including H85, demonstrated *APC*-specific copy number variation (duplication or deletion) in their sequenced polyp DNA. (**C**) Copy number variations detected through sequencing across the panel of 283 target genes by patient: none = 0 CNVs (4/19), few = 1–4 (9/19), moderate = 5–25 (3/19), extensive = >26 (4/19). (**D**) *APC* mRNA sequence of normal and mutant alleles in patients H72 and H73 at the familial mutation site (c.5826_5829). The highlighted CAGA sequence is deleted in the germline mutant *APC* allele. (**E**) Chromatogram of sequenced *APC* cDNA harvested from H72 polyp organoids grown in 100% WR media, H72 polyp organoids grown for 2+ passages in WR-free media, and H73 polyp organoids grown for 3+ passages in WR-free media, depicting 7 bp 5′ of the mutation site and 6 bp 3′ of the mutation site. The blue underlined sequence aligned with the wild-type sequence. The red underlined sequence aligned with the mutant sequence. (**F**) *APC* mRNA sequence of the wild-type and mutant alleles in patient H87 at the familial mutation site (c.4782_4785). The highlighted AGCC sequence is deleted in the germline mutant *APC* allele. (**G**) Chromatogram of sequenced *APC* cDNA harvested from H87 polyp organoids grown in WR media or for 2+ passages in WR-free media. The blue underlined sequence aligned with the wild-type sequence. The red underlined sequence aligned with the mutant sequence.

We sought to understand the mutational landscape that led to enhanced Wnt signaling in biopsies and organoids that may underscore FGP formation. One likely mechanism would be somatic *APC* mutation leading to loss of heterozygosity and further upregulation of the Wnt signaling pathway as this underscores FAP polyp emergence in the colon. DNA was extracted from our initial 19 patient biopsies or organoids for genomic analysis of tumor suppressors and oncogenes using a QIAGEN Human Comprehensive Cancer Panel, which included *APC* (Table 2). In each paired patient set (P vs. NP), we verified the familial *APC* mutation. Our analysis revealed that only 1 of the 19 patients studied (H85) had a novel somatic loss-of-function *APC* mutation (Figure 4A and Table 2). Further intrapatient comparisons revealed that about one-third (6/19) of polyp samples harbored *APC-*specific copy number variations, though this analysis did not provide insight into whether the mutant or functional (normal) allele was affected (Figure 4B and Table 2). Looking broadly at the genomic landscape across the target genes included in the panel, we observed allelic variation that demonstrated chromosomal changes ranging from no observed copy number variations to extensive (50+) incidences in polyps, suggesting enhanced chromosomal instability was frequently associated with polyposis (Figure 4C and Table 2).

### A low-Wnt environment can select for Wnt-activating mutations to sustain organoid growth.

We tested whether there was transcriptional silencing of the wild-type *APC* allele during polyp organoid culture as another mechanism to enhance Wnt tone. *APC* is hypermethylated in gastric cancer (53%) relative to normal (38%) tissue (29). Although high-Wnt polyp organoids from H72, H73, and H87 demonstrated few genomic mutations or copy number variations, transcriptional silencing of the wild-type allele could explain their elevated Wnt tone. We tested whether high-Wnt organoids grown in WR or WR-free media exhibited loss of the wild-type *APC* transcript by sequencing mRNA harvested from these 3 organoid lines at their respective *APC* familial mutation.

Patients H72 and H73, a parent-child pair sharing a common familial mutation, and patient H87 had inherited 4 bp deletions at c.5826_5829 and c.4782_4785, respectively (Figure 4, D and F). Sequencing *APC* mRNA at the familial mutation site for H72 and H73 polyp-derived organoids grown in WR culture conditions verified transcription of both normal and mutant alleles (Figure 4E and Supplemental Figure 6). This was determined by bifurcation of sequencing products 3′ to the mutation site. However, after growth for 3 passages in WR-free media, H72P and H73P organoids only expressed the mutant allele (Figure 4E). In contrast, H87P organoids retained the wild-type *APC* transcript in both growth conditions (Figure 4G).

To further understand the dynamics of loss of wild-type *APC* in H73P organoids grown in WR-free media, we used organoids from the earliest available passage after initial biopsy seeding to minimize the influence of genetic drift in culture. H73P organoid RNA was harvested at passage 2 in WR media and then at passage 3 after 6 days’ growth in 100%, 50%, or 0% WR media (Supplemental Figure 6A). As before, we detected both wild-type and mutant transcripts in WR conditions (Supplemental Figure 6B). However, after passage, while organoids grown in 100% or 50% WR media maintained expression of both alleles (Supplemental Figure 6C), growth in WR-free conditions rapidly led to loss of the wild-type transcript. Thus, although increased Wnt tone in high-Wnt organoids was not associated with silencing of the normal *APC* allele when grown in media containing WNT/RSPO ligands, organoid growth in a WR-free environment can select for loss of expression of the wild-type *APC* allele.

Following the “just-right” hypothesis, we hypothesized that the optimal growth of polyp organoids adapted to WR-free culture conditions would occur in media with minimal exogenous Wnt. We titrated each of the 3 WR-free lines to their optimal Wnt signaling environment and determined that optimal growth occurred in 20% WR media (Supplemental Figure 6D). Ultimately, this establishes a relationship between extrinsic and intrinsic Wnt in accordance with the “just-right” hypothesis for gastric corpus organoids. As intrinsic Wnt tone is increased through mutation or otherwise, tolerance to extrinsic Wnt is reduced (Supplemental Figure 6E).

### Mice demonstrate region-specific gastric proliferation in response to Apc mutation.

Our data suggest that genomic *APC* loss of heterozygosity is not required for FAP polyp emergence. Further, FAP organoid growth suggests that polyp organoids do not tolerate high levels of extrinsic or pharmacologic Wnt activation in accordance with the “just-right” hypothesis. These findings, in combination with the low clinical prevalence of cancer progression from FAP polyps, suggest that additional somatic mutations to *APC*, such as *APC-*null, may not be tolerated within the corpus epithelium. Rather, in the corpus, the *APC*-heterozygous state may be sufficient to promote growth. We sought to test these concepts in vivo in a mouse FAP model to determine the effect of genomic *Apc* loss on corpus epithelial cell proliferation compared with the antrum, where, in patients with FAP, infrequent polyps are typically adenomatous.

**Figure 5:**
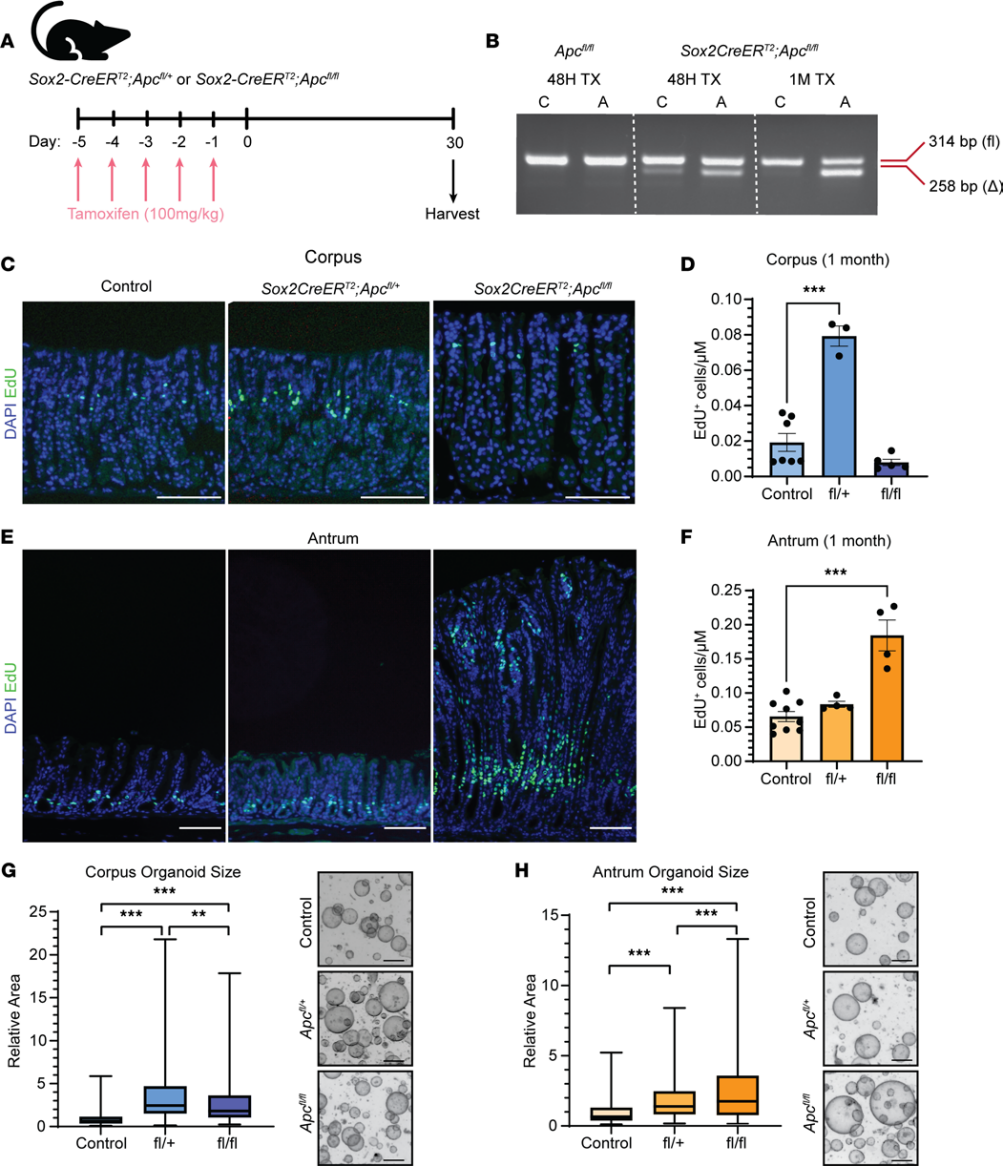
Gastric region–specific proliferation in FAP mouse model. (**A**) Adult *Sox2-CreER^T2^ Apc^fl/+^* (heterozygous), *Sox2-CreER^T2^ Apc^fl/fl^* (homozygous), and control (*Apc^fl/+^* or *Apc^fl/fl^*) mice were treated with tamoxifen (TX) to delete *Apc* exon 14, and tissue was harvested 1 month later. (**B**) Agarose gel showing PCR products amplified from genomic DNA with primers flanking a *loxP* site in the *Apc* gene to identify either the un-recombined allele (fl, 314 bp) or recombined allele (Δ, 258 bp). DNA was isolated from full-thickness corpus (C) or antral (A) tissue from control (*Apc^fl/fl^*) mice 1 month post-TX or from homozygous *Sox2CreER^T2^ Apc^fl/fl^* mice either 48 hours or 1 month post-TX. (**C** and **E**) Representative images of corpus (**C**) and antral (**E**) tissue from control, *Sox2-CreER^T2^ Apc^fl/+^*, and *Sox2-CreER^T2^ Apc^fl/fl^* mice 1 month post-TX stained for 5-ethynyl-2′-deoxyuridine (EdU) (green) to mark proliferating cells (scale bar = 100 μm). (**D** and **F**) Morphometric quantification of proliferating EdU^+^ cells/μm of corpus (**D**) or antral (**F**) tissue in control, heterozygous (fl/+), and homozygous (fl/fl) mice 1 month post-TX. Data are presented as mean ± SEM (*n* = 3–9 mice per group, ***P < 0.001 by 1-way ANOVA with Tukey’s multiple-comparison test). (**G** and **H**) Size and representative images of corpus (**G**) or antral (**H**) organoids derived from control (*Sox2-CreER^T2^*), heterozygous (fl/+), and homozygous (fl/fl) mice. Data are presented as fold-change organoid area relative to control (error bars minimum to maximum values, box length IQR, whisker outliers) (**P < 0.05, ***P < 0.005 by 1-way ANOVA with Tukey’s multiple-comparison test). *Sox2-CreER^T2^* (*n* = 3 mice; 208 corpus organoids; 217 antrum organoids), *Sox2-CreERT2 Apc^fl/+^* (*n* = 3 mice; 171 corpus organoids; 256 antrum organoids), and *Sox2-CreERT2* *Apc^fl/fl^* (*n* = 3 mice; 218 corpus organoids; 278 antrum organoids).

*Apc^fl^* mice with *loxP* sites surrounding exon 14 were crossed to *Sox2-CreER^T2^* mice. *Apc* mutation was induced in adults by treatment with tamoxifen, and gastric tissue was harvested from control (*Apc^fl/+^* or *Apc^fl/fl^*), heterozygous (*Sox2-CreER^T2^* *Apc^fl/+^*), and homozygous (*Sox2-CreER^T2^ Apc^fl/fl^*) mice 1 month later (Figure 5A). We confirmed *Apc* exon 14 deletion in DNA isolated from corpus and antrum by PCR amplification 48 hours post-TX (Figure 5B). Further analysis demonstrated that heterozygous *Apc* deletion was maintained 1 month postdeletion in both corpus and antrum while, in contrast with antrum, homozygous deletion of *Apc* was poorly maintained in the corpus (Figure 5B and Supplemental Figure 7).

Further analysis of tissue histology and epithelial cell proliferation also showed stark differences in the cellular response to *Apc* mutation in the gastric corpus and antrum. While *Apc* homozygous deletion had no apparent effect on corpus tissue 1 month post-TX (Figure 5), polyp-like growths were observed throughout the antrum (Supplemental Figure 8). Strikingly, while morphometric analysis of proliferation showed no change in corpus epithelial proliferation in homozygous mice 1 month post-TX, we observed a significant (~4×) increase in heterozygous mice (Figure 5, C and D). In contrast, there was no observed change in antral proliferation in heterozygous *Apc*-mutant mice whereas homozygous *Apc* mutation resulted in profound hyperproliferation and the formation of polyp-like structures (Figure 5, E and F, and Supplemental Figure 8).

We further tested the effect of *Apc* mutation on mouse gastric organoid growth. Organoids were initiated from gastric glands isolated from control, heterozygous, and homozygous *Apc*-mutant mice 48 hours post-TX treatment, and organoid size was measured 4 days later. Consistent with the in vivo findings, heterozygous *Apc*-mutant corpus organoids (*Sox2-CreER^T2^* *Apc^fl/+^*) grew larger than control while homozygous *Apc*-deleted corpus organoids (*Sox2-CreER^T2^ Apc^fl/fl^*) exhibited diminished growth (Figure 5G). In contrast, antral organoids demonstrated a stepwise growth advantage in response to heterozygous and homozygous *Apc* deletion (Figure 5H).

## Discussion

This study investigated how dysregulation of Wnt signaling in patients with FAP leads to abundant yet benign FGPs in the gastric corpus. We initially hypothesized that polyp emergence in the FAP patient corpus was due to somatic mutation resulting in *APC* loss of heterozygosity. This would align with the general understanding of colorectal polyposis in which a second hit to *APC* is considered a requirement and the first step in the mutational cascade inevitably leading to colorectal cancer (18, 30). Surprisingly, we determined that *APC* loss of heterozygosity is not a requirement for gastric FGP formation in patients with FAP as underlying somatic *APC* alteration was uncommon. A prior comparative study of the mutational landscape underscoring FGP formation demonstrated that 51% (21/41) of FAP-associated polyps obtained from 17 patients had acquired a somatic *APC* gene alteration through either a loss-of-function mutation (15/41) or allelic loss (6/41) resulting in loss of heterozygosity (31). Although the prior study did demonstrate a higher proportion of FGPs arising from somatic *APC* gene alterations than ours, it aligns with our finding that *APC* loss of heterozygosity is not a requirement for their emergence.

Despite an absence of a second-hit somatic *APC* alteration within our FAP patient biobank, we observed that Wnt signaling was generally upregulated in FGP biopsy samples as well as within a subset of our polyp-derived organoids. This underscores a role for Wnt activation in driving polyp formation; however, a precise mechanism driving increased pathway activation within these polyps remains unclear. We explored *APC* copy number variation and transcriptional regulation as 2 mechanisms that could lead to increased Wnt signaling in lieu of genomic mutation. Although we did not observe transcriptional silencing of the normal *APC* allele in organoids under normal growth conditions, we did observe that some lines were capable of acquiring this phenotype in order to survive within a WNT/RSPO-free environment. Further, our targeted genomic analysis demonstrated *APC* copy number variation within some FGPs (6/19), as well as significant copy number variation across our full gene panel. This finding aligns with previous studies in colorectal cancer cell lines showing that loss of APC function leads to chromosomal instability by disrupting its role in microtubule binding (32). We speculate that heterozygous loss of *APC* confers genetic instability that could sensitize the tissue to polyposis in lieu of a second-hit *APC* mutation.

Alterations to other key regulators of the Wnt pathway, such as β-catenin, could explain increased Wnt signaling in FAP polyps. While we did not observe *CTNNB1* mutations within our data set, studies have shown that sporadic FGPs in otherwise-healthy patients often contain β-catenin activating mutations, further supporting a role for elevated Wnt signaling in FGP formation (33, 34). Overall, we hypothesize that the mechanisms leading to enhanced intrinsic Wnt signaling within FGPs are likely complex and vary from polyp to polyp as we observed substantial variability in Wnt responses across our patient set and even within single patients.

Our biobank of FAP polyp-derived organoids included some without apparent increases in intrinsic Wnt signaling as they exhibited similar gene expression and Wnt tolerance as NP organoids. Notably, these low-Wnt or P-N organoids were derived from a variety of patients with FAP without obvious associations with *APC* genotype or clinical features such as proton pump inhibitor usage, gastric polyp phenotype, or intestinal polyp burden (Table 1 and Supplemental Table 1). It is plausible that outgrowth of cells with more normal-like Wnt characteristics could be selected for in vitro because all organoids were established in media containing WNT/RSPO, therefore providing a growth advantage toward cells with more moderate Wnt characteristics. However, primary tissue biopsies also demonstrated heterogeneity in Wnt target gene expression, suggesting that selection in culture does not necessarily underlie this variation in intrinsic Wnt tone among our organoid collection. Therefore, additional Wnt-independent mechanisms likely also underscore FGP emergence in patients with FAP. A recent study of wild-type murine gastric organoids utilized a genome-scale CRISPR screen to identify genes promoting growth in WNT/RSPO-depleted conditions (35). While mutations to Wnt pathway genes, such as *Apc* and *Alk*, were found to support organoid growth by upregulating intrinsic Wnt signaling, this screen also identified genes, including *Bclaf3* and *Prka*, that supported growth through Wnt-independent mechanisms. Additional mechanisms, such as through environmental factors, may also play a role in the manifestation of FGPs in patients with FAP without a requirement for Wnt pathway upregulation.

Regardless of the mechanism by which Wnt is upregulated in FGPs in patients with FAP, the same underlying *APC* mutation appears to have strikingly different effects in driving polyposis in the human gastric corpus versus antrum. Wnt signaling has been demonstrated to play a critical role in differentially regulating identity between the corpus and antral compartments, with high signaling levels being essential for corpus specification during development (36, 37). A recent study from our lab demonstrated that human corpus-derived organoids generated from patients without underlying disease have a reduced threshold for Wnt to induce growth compared with patient-matched antral organoids (24). Combined with the present study, our findings suggest that corpus polyposis in patients with FAP may result from an intrinsic heightened sensitivity for Wnt to drive proliferation in corpus progenitor cells. That is, the familial, heterozygous *APC* mutation may prime corpus progenitors to hyperproliferate, while antral progenitors require further second-hit *APC* mutations to induce proliferation, thus underscoring the increased risk of antral adenoma. This aligns with the abundance of FGPs (hundreds to thousands of lesions) in some patients with FAP, as there would be no requirement for the rare genomic occurrence of a second *APC* hit. Comparative studies of corpus FGPs and antral polyps in patients with FAP would provide further context regarding the underlying genetic mechanisms underscoring emergence and potential for progression to cancer. However, antral polyps are rare in patients with FAP, and the increased malignant potential of the few that do arise complicates their acquisition for research purposes. Therefore, we were unable to obtain antral polyps during this study to conduct these interesting comparative analyses.

Our hypothesis of regional responses to Wnt tone underscoring polyp emergence was supported by analysis of an FAP mouse model, where we showed stark differences in response to *Apc* mutation in the corpus and antrum. We observed that the murine stomach displayed a similar regional pattern as human FAP, with heterozygous *Apc* deletion (*Apc^+/–^*) promoting corpus proliferation and homozygous deletion (*Apc^–/–^*) promoting profound antral hyperplasia while the corpus is spared. Our data showed that the homozygous *Apc* mutation was not maintained in the corpus, thus suggesting that loss of heterozygosity is not tolerated in this gastric region. Previous studies in mice have also shown that homozygous deletion of *Apc* in differentiated chief cells did not promote corpus hyperplasia (38, 39). Additionally, a long-term study of an *Apc*^min/+^ mouse model, which closely mimics the FAP condition through a germline *Apc* mutation, demonstrated that gastric tumors in these mice overwhelmingly arise within the antrum while only rarely occurring in the corpus (40). The majority of these antral tumors were verified to be adenomas resulting from *Apc* loss of heterozygosity. Our study contextualizes these findings by demonstrating that *Apc^–/–^* cells either are not retained in the corpus epithelium long term or do not contribute to cellular turnover. Conversely, *Apc^+/–^* cells are maintained and contribute to enhanced corpus proliferation. Importantly, another study of heterozygous *Apc*-mutant mice (*Lrig1-CreER^T2^ Apc^fl/+^*) showed evidence of hyperplasia and increased proliferation in the corpus and antrum 100+ days following recombination (41). Overall, the body of literature in combination with our results suggest that heterozygous loss of *Apc* in mice mimicking the FAP condition promotes a favorable environment for corpus proliferation while additional *Apc* gene mutation is not tolerated.

Our findings align with a principle of Wnt signaling known as the “just-right” hypothesis, which describes a Goldilocks zone of signaling tone to drive excessive growth in the context of FAP polyposis. Studies exploring this concept have demonstrated regional differences in Wnt signaling tone throughout the small intestine and colon that contribute to region-specific Wnt sensitivity and tumorigenesis (20, 23, 27, 42). We have now expanded this understanding to the stomach by demonstrating that regional differences in Wnt signaling in the corpus versus the antrum may contribute to the emergence of gastric polyps.

The “just-right” hypothesis predicts an upper limit to Wnt signaling that becomes growth prohibitive (22, 43). An intermediate level of Wnt signaling, therefore, best promotes hyperproliferation and tumorigenesis, with the optimal level differing along the gastrointestinal tract (20, 22, 43). Our study verifies these findings in gastric polyp organoids and demonstrates that increased intrinsic Wnt signaling sensitizes organoids to additional upregulation of the Wnt pathway. This, again, aligns with the concept that somatic *APC* mutation may not be tolerated within the FAP corpus in vivo as Wnt signaling would be elevated beyond an upper threshold of tolerability. Furthermore, these principles would underscore the benign nature of FGPs relative to polyps of the antrum and colon.

In conclusion, our study translates the “just-right” hypothesis of Wnt signaling to the clinical manifestation of gastric disease caused by pathway dysregulation and develops 2 conclusions related to FGP emergence and pathogenesis in patients with FAP. First, we demonstrate that Wnt signaling is predominately upregulated in polyp tissues as well as in polyp-derived organoids and therefore validate that enhanced intrinsic Wnt signaling is a key characteristic of FGPs. The lack of requirement for a second hit to *APC* may explain the abundant manner in which FGPs emerge. Second, we establish a definitive relationship between intrinsic and extrinsic Wnt signaling in gastric corpus organoids and demonstrate that additional Wnt signaling in cells with intrinsically upregulated Wnt tone prohibits growth. The corpus environment may therefore selectively prohibit high intrinsic Wnt signaling, such as through *APC* loss of heterozygosity, thus underscoring the benign nature of FGPs as well as the regionality of their emergence.

## Methods

### FAP patient biopsy collection and processing.

Human gastric tissue biopsies were collected from patients undergoing endoscopy at Michigan Medicine by experienced endoscopists who were familiar with the design and goals of the study. Biopsies were obtained using cold forceps to target areas of polyp tissue and tissue without visible polyps. Biopsies were placed in 15 mL conical tubes containing 5 mL of ice-cold DPBS (Gibco, 14190144) with antibiotic-antimycotic (100 U/mL Pen/Strep + 250 ng/mL Amphotericin B, Gibco, 15240062) for transport to the research lab.

Biopsies were minced into small fragments (~1 mm) and divided for nucleotide (DNA or RNA) extraction or organoid formation. Fragments set aside for nucleotide extraction were placed into 1.5 mL Eppendorf tubes and snap-frozen in liquid N_2_. Fragments for organoid formation were transferred to 5 mL ice-cold DPBS + antibiotic-antimycotic for subsequent processing. In some instances, minced biopsies were frozen for later organoid development by resuspending in DMEM/F12 (Gibco, 12634010) with 10% DMSO and 10% fetal bovine serum (MilliporeSigma, F0926) and frozen in cryovials for long-term storage in liquid N_2_ (44).

### Establishment and culture of human gastric organoids.

Organoids were established from isolated gastric glands or directly from minced tissue that was embedded in 40 μL Matrigel (Corning, 354234) in a 24-well plate. For the isolated-gland approach, 15 mM EDTA (Invitrogen, 15575038) was added to minced biopsies in 5 mL DPBS + antibiotic-antimycotic in 15 mL conical tubes and rocked at 4°C for 1 hour. Tissue fragments were transferred to a new tube containing 8 mL of ice-cold DPBS, and gastric glands were released by vortexing at maximum speed for 2 minutes. The suspended gastric glands were transferred to a new tube, pelleted at 600*g* for 5 minutes at 4°C, and resuspended in Matrigel for plating. Alternatively, minced tissue was directly embedded in Matrigel, with epithelial outgrowths emerging within 2–3 days of plating (Supplemental Figure 3). Organoid lines were established by passaging at least 3 times before analysis.

Organoids were maintained in WR (WNT, RSPO; Noggin), WR-free (Noggin), or CN (CHIR99021, Noggin) media as specified. 100% WR media were generated with 50% L-WRN conditioned media (University of Michigan Translational Tissue Modeling Laboratory), 10% fetal bovine serum (MilliporeSigma, F0926), 37% DMEM/F12 (Gibco, 12634010), 2 mM GlutaMAX (Gibco, 35050061), Antibiotic-Antimycotic (100 U/mL Pen/Strep + 250 ng/mL Amphotericin B, Gibco, 15240062), 10 μM Y-27632 (Tocris, 1254), 10 μM SB431542 (Tocris, 1614), and 50 μg/mL Gentamycin (Gibco, 15750060). WR-free media consisted of 20% fetal bovine serum, 77% DMEM/F12, GlutaMAX, Antibiotic-Antimycotic, Y-27632, SB431532, and Gentamycin in the same concentrations as above and were supplemented with 100 nM recombinant Noggin (R&D Systems, 6057-NG). For WR media formulations, normal (100%) WR and WR-free media were mixed at appropriate ratios to obtain the desired final WR concentration. For CN media, CHIR (Tocris, 4423) was added to WR-free media to obtain the final concentration as specified. DMSO added through stock solutions of CHIR and SB431542 was maintained at a final concentration of 0.3% in CN media.

Organoid cultures were maintained by replenishing with fresh media every 2 days and passaging every 6 days. To passage organoids, media were aspirated, and Matrigel patties containing organoids were overlaid with 500 μL of cold DPBS, mechanically disrupted through scraping with a P1000 pipet tip, and transferred to a 1.5 mL Eppendorf tube. Organoids were pelleted at 250*g* for 5 minutes at 4°C, resuspended in 500 μL of TrypLE Express (Gibco, 12604013), and incubated at 37°C for 10 minutes. Organoids were vigorously pipetted approximately 40 times with a P1000 to dissociate into single cells. A total of 700 μL of cold DPBS was added, cells were pelleted at 250*g* for 5 minutes at 4°C, cells were resuspended in 30–100 μL of DMEM, and cell concentration was quantified using a hemocytometer. For all experiments, cells were plated at a density of 300 cells/μL of Matrigel patty. Matrigel patties were given 30–45 minutes to solidify at 37°C, then overlaid with the appropriate warmed media.

### DNA library preparation.

DNA was extracted from FAP patient biopsies using the QIAGEN Blood & Tissue DNA kit, with sample elution in 100–200 μL Buffer TE. For DNA extraction from paired NP and P organoid samples, culture media were aspirated from each Matrigel patty (3 wells pooled/sample); organoids were suspended in DPBS, pelleted at 300*g* for 5 minutes, and resuspended in 200 μL DPBS; and DNA was extracted.

For preparation of genomic DNA libraries, samples were indexed using the QIAseq 96-Index I Set A kit (QIAGEN 333727), and libraries were constructed for sequencing using the QIAseq targeted Human Comprehensive Cancer Panel (QIAGEN 333515, catalog DHS-3501Z-96). Next-generation DNA sequencing was performed using a HiSeq 4000 (Illumina), with sequencing and analysis conducted in collaboration with the University of Michigan Advanced Genomics Core.

### RNA extraction, qPCR analysis, and mRNA sequencing.

RNA was isolated using the QIAGEN Mini Kit according to the manufacturer’s instructions. Frozen minced tissue from biopsies was transferred to RLT Buffer + 1% βME and homogenized, then centrifuged at 14,000*g* for 3 minutes at 4°C to pellet particulates. Supernatant was collected, transferred to a new tube, and mixed with an equal volume of 70% EtOH. For organoid RNA extraction, organoids were dispersed to single cells using TrypLE, washed, resuspended in RLT Buffer + 1% βME, and vortexed at max speed for 30 seconds before RNA extraction.

cDNA was synthesized from 250 ng RNA using the iScript cDNA Synthesis Kit (BioRad, 1708891). qPCR reactions used the iTaq Universal SYBR Green Supermix (BioRad, 1725124) and respective primers (Supplemental Table 2). qPCR was performed with samples in triplicates, and average cycle threshold values were quantified relative to the reference mRNA *ACTB* or *HPRT* using the ΔΔCT method to determine mRNA abundance.

For *APC* mRNA sequencing, RNA was extracted from organoids, cDNA was synthesized, and qPCR reactions were performed using primers encompassing the *APC* mutation site (Supplemental Table 3). PCR products were purified using the QIAquick PCR Purification Kit according to the manufacturer’s instructions, then sent to Eurofins Genomics for sequencing, and sequences were analyzed using SnapGene software.

### Coculture and conditioned media experiments.

P organoids from lines H72, H73, and H87, as well as NP organoids from H87, were dispersed to single cells. For coculture experiments, single cells from P and NP organoids were plated in independent 10 μL patties of Matrigel at a density of 3,000 cells/patty within the same well of a 24-well plate, taking care to ensure patties did not touch. For conditioned media experiments, the 3 high-Wnt organoid lines were each plated in independent 10 μL patties of Matrigel at a density of 3,000 cells/patty within the same well of a 24-well plate. Cells were overlaid with WR-free media. On day 4, media from high-Wnt P organoids were transferred to patties containing freshly passaged single cells from NP organoids. WR-free media were replenished, and this was repeated every 2 days for a total of 6 days.

### Human organoid growth experiments.

For Wnt titration experiments, organoids were dispersed to single cells, plated into triplicate wells of a 24-well plate in 40 μL patties of Matrigel at a density of 12,000 cells/well, and overlaid with appropriate media. On day 6, triplicate wells of each media condition were consolidated, organoids were dispersed to single cells, and cells were resuspended at the same split ratio across all conditions. The concentration of cells in 100% WR was calculated, and cells from each condition were plated at the split ratio calculated to reach 12,000 cells/well in 40 μL Matrigel patties for the 100% WR condition. For measurement of growth, media were aspirated, and patties were overlaid with a room temperature 50:50 mixture of DMEM (200 μL) and CellTiter-Glo 3D (200 μL) (Promega, G9681). Plates were incubated for 30 minutes at room temperature in the dark, the Matrigel patty was broken down by rapid pipetting, and the total contents of each well were transferred to individual wells of a white, opaque, 96-well plate. Luminescence was measured using a plate reader (Spectra Max M5e, Molecular Devices) with an integration time of 500 ms.

For CHIR growth experiments, organoids were dispersed to single cells and plated into a 96-well, round-bottom plate in 5 μL patties of Matrigel at a density of 1,500 cells/well. For measurement of growth, media were aspirated, and 150 μL of a room temperature 50:50 mixture of DMEM (75 μL) and CellTiter-Glo 3D (75 μL) (Promega, G9681) was added to each well. Plates were incubated for 30 minutes at room temperature in the dark, the Matrigel patty was broken down by rapid pipetting, and the total contents of each well were transferred to individual wells of a white, opaque, 96-well plate. Luminescence was measured using a plate reader with an integration time of 500 ms.

### Mouse experiments.

Adult mice of both sexes 2–3 months old were housed under specific pathogen–free conditions. *Sox2-CreER^T2^* (Jackson Laboratory 017593) (45) and *Apc580^fl^* (*Apc^fl^*) (46) mice have been previously described. *Apc^fl^* mice contain a floxed exon 14, which upon Cre-mediated deletion encodes an APC protein truncated at codon 580. *Apc^fl^* mice were on a 129/SvJae background, and *Sox2-CreER^T2^* mice were on a mixed C57BL/6 129/SvJae background. *Sox2-CreER^T2^* and *Apc^fl^* mice were bred to generate the following genotypes: *Sox2-CreER^T2^* *Apc^fl/+^* (heterozygous), *Sox2-CreER^T2^ Apc^fl/fl^* (homozygous), and control (*Apc^fl/+^* and *Apc^fl/fl^*, or *Sox2-CreER^T2^*).

To induce Cre-mediated recombination of the floxed *Apc* allele, mice were treated with 100 mg/kg TX via intraperitoneal injection once daily for 5 days. For analysis of *Apc* recombination, full-thickness tissue was homogenized, and DNA was isolated using the DNeasy Blood & Tissue Kit (QIAGEN, 69504) per the manufacturer’s instructions. Recombination was analyzed via PCR amplification using the following primers (46): P3: 5′-GTTCTGTATCATGGAAAGATAGGTGGTC-3′, P4: 5′-CACTCAAAACGCTTTTGAGGGTTGATTC-3′, P5: 5′-GAGTACGGGGTCTCTGTCTCAGTGAA-3′. P3 and P4 generate a 314 bp product encompassing the un-recombined *loxP* site, while P3 and P5 generate a 258 bp product encompassing the recombined region.

For analysis of proliferation, mice were injected with EdU (25 mg/kg, Invitrogen, A10044) 2 hours prior to tissue collection. For morphometric quantification of EdU incorporation, the entire length of the corpus and antrum for each animal was imaged (*n* = 3–9 animals per group), and blinded cell counts were normalized to epithelial tissue length (μm) using ImageJ software (NIH).

### Analysis of murine gastric organoids.

Mouse gastric organoids were established from corpus and antrum tissue collected from *Sox2-CreER^T2^* (control), *Sox2-CreER^T2^ Apc^fl/+^* (heterozygous), and *Sox2-CreER^T2^ Apc^fl/fl^* (homozygous) mice 48 hours after final TX injection. Gastric tissue was washed in ice-cold DPBS and minced into fragments of 2–3 mm. Tissue fragments were transferred to DPBS containing 8 mM EDTA and rocked at 4°C for 1 hour. To isolate glands, tissue fragments were transferred to a fresh DPBS solution containing 10 mM EDTA and rocked at 4°C for 2 hours. During the last 10 minutes of incubation, samples were placed on ice to allow glands to settle, and the EDTA-DPBS solution was replaced with DPBS and gently mixed 3–5 times using a fetal bovine serum–coated p1000 tip with the opening enlarged to prevent breaking or sticking of isolated glands. Once the mixture was cloudy, glands were transferred to a 1.5 mL Eppendorf tube and centrifuged at 150*g* for 10 minutes at 4°C. After aspirating the supernatant, glands were resuspended in 40 μL Matrigel and plated. Murine gastric organoids were maintained in culture using the same procedures outlined for human organoid growth but without the TGF-β inhibitor SB431542.

For size measurement, mouse organoids were imaged at day 4 of culture using an Olympus stereomicroscope at 1.7× original magnification. The area of growth was calculated from digital images using the Orgaquant software (47). Three technical replicates were used for each group and were pooled for analysis, with more than 170 organoids measured from each condition.

### Statistics.

GraphPad Prism (version 9.0) was used for statistical analysis. Student’s *t* test (2 tailed) was used to compare 2 groups. For comparison of 3 or more groups, a 1-way ANOVA was used followed by a Tukey post hoc test. *P* < 0.05 denoted significance.

### Study approval.

Collection of human tissue was conducted under Institutional Review Board–approved protocol (HUM00102771) at the University of Michigan. Written informed consent was provided by individual patients prior to collection of biopsies. Mouse studies were conducted under University of Michigan Institutional Animal Care & Use Committee–approved protocols (PRO00010803).

### Data availability.

Underlying data, including annotated sequencing data, are available in the corresponding Supporting Data Values file. Organoid lines are available upon reasonable request to the corresponding author.

## Author contributions

KPM, ED, TMK, ESH, and LCS were responsible for designing research studies. KPM, ED, and TMK conducted experiments and acquired data. KPM, ED, TMK, and LCS were responsible for analyzing data. DKT and EMS were responsible for coordinating human biopsies and providing clinical information. KPM and LCS were responsible for writing the manuscript, and all other authors read and provided comments on the manuscript.

## Supplementary Material

Supplemental data

Supporting data values

## Figures and Tables

**Table 1:**
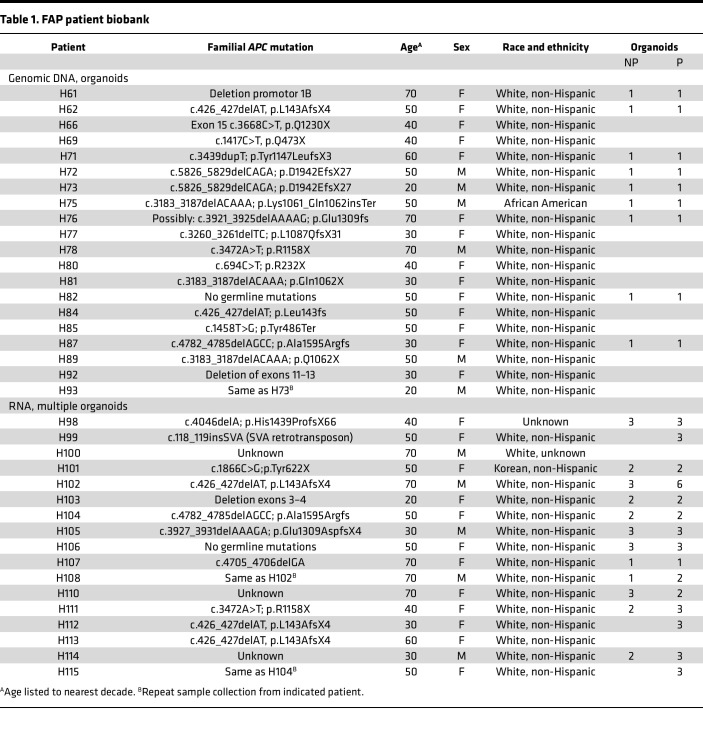
FAP patient biobank

**Table 2:**
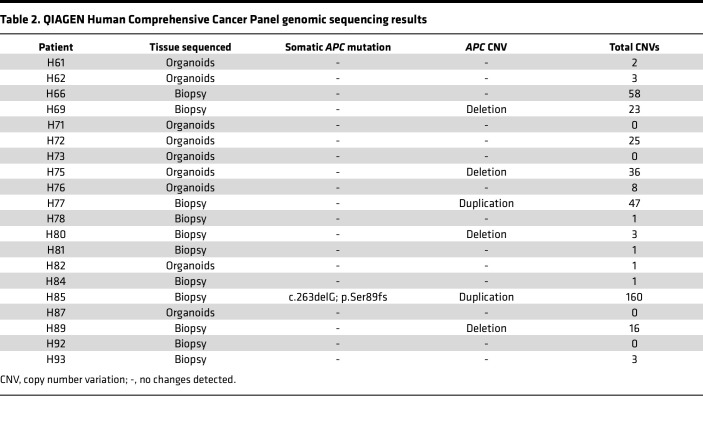
QIAGEN Human Comprehensive Cancer Panel genomic sequencing results

## Version 1 11/09/2023

In-Press Preview

## Version 2 12/22/2023

Electronic publication
