# Supplementary material for: Region-specific Wnt signaling responses promote gastric polyp formation in patients with familial adenomatous polyposis
Source: JCI Insight. 2023 Nov 9;8(24):e174546. doi: 10.1172/jci.insight.174546 (PMC10896006; doi:10.1172/jci.insight.174546)
Supplement: Supplemental data [file jciinsight-8-174546-s020.pdf]

Supplementary Information

Region-specific Wnt signaling responses promote gastric polyp formation in familial adenomatous polyposis patients

Kevin P. McGowan[1], Elizabeth Delgado[1], Theresa M. Keeley[1], Elise S. Hibdon[1], D. Kim Turgeon[2], Elena M. Stoffel[2], Linda C. Samuelson[1,2*]

Departments of [1]Molecular & Integrative Physiology, and [2]Internal Medicine, University of Michigan, Ann Arbor, MI

Supplementary Table 1: Clinical gastrointestinal phenotype of FAP patients.
Supplementary Table 2: Oligonucleotide primer sequences used for qRT-PCR gene expression analysis.
Supplementary Table 3: Oligonucleotide primer sequences used for *APC* mRNA amplification.
Supplementary Figure 1: mRNA expression analysis of primary FGP and surrounding non-polyp biopsies from FAP patients.
Supplementary Figure 2: High Wnt FAP organoids are not sustained through secreted ligands.
Supplementary Figure 3: Establishment of human FAP gastric organoids from biopsies.
Supplementary Figure 4: Polyp-derived FAP organoids exhibit significant intra-patient variability in Wnt dependence.
Supplementary Figure 5: Gene expression in FAP organoids.
Supplementary Figure 6: WR-Free media selects for organoids with transcriptional loss of wildtype *APC* expression.
Supplementary Figure 7: Heterozygous loss of *Apc* is maintained long-term within the corpus epithelium.
Supplementary Figure 8: Antral polyps develop in FAP mouse model with homozygous *Apc* mutation.

Supplementary Table 1: Clinical gastrointestinal phenotype of FAP patients.

	Patient	Gastric Polyp Phenotype			PPI Usage	Duodenal Phenotype*	Colorectal Phenotype
		Number	Size	Dysplasia			
Genomic DNA, Organoids	**H61**	Multiple	2-6 mm	-	-	1	Classic
	**H62**	50+	5-8 mm	LGD	-	0	Attenuated
	**H66**	20+	2-5 mm	-	-	2	Classic
	**H69**	-	3-6 mm	LGD	-	2	Classic
	**H71**	20+	3-5 mm	LGD	-	-	Classic
	**H72**	100+	2-3 mm	-	-	0	Attenuated
	**H73**	100+	-	-	No	0	Attenuated
	**H75**	<10	3-4 mm	HGD	Yes	3	Classic
	**H76**	Multiple	3-6 mm	-	Yes	3	Classic
	**H77**	10+	3-5 mm	-	Yes	0	Classic
	**H78**	Multiple	3-6 mm	-	Yes	2	Classic
	**H80**	Multiple	-	-	Yes	0	Classic
	**H81**	15+	2-3 mm	-	Yes	0	Classic
	**H82**	Multiple	3-6 mm	-	No	3	Classic
	**H84**	100+	3-9 mm	-	No	1	Classic
	**H85**	Multiple	4-10 mm	IM	No	2	Attenuated
	**H87**	Multiple	1-3 mm	-	Yes	0	Attenuated
	**H89**	<10	Small	-	No	2	Classic
	**H92**	Multiple	1-4 mm	-	Yes	2	Classic
	**H93**	100+	-	-	No	0	Classic
RNA, Multiple Organoids	**H98**	-	-	-	-	-	-
	**H99**	Carpeting	-	-	Yes	1	Attenuated
	**H100**	Multiple	3-8 mm	-	Yes	3	Classic
	**H101**	10+	2-4 mm	-	Yes	0	Classic
	**H102**	Carpeting	2-5 mm	-	Yes	0	Classic
	**H103**	Carpeting	3-9 mm	LGD	No	0	Attenuated
	**H104**	Carpeting	-	-	Yes	0	Classic
	**H105**	30+	3-6 mm	LGD	Yes	1	Classic
	**H106**	20+	2-3 mm	-	Yes	2	Classic
	**H107**	Carpeting	-	LGD	No	0	Classic
	**H108**	Carpeting	2-5 mm	-	Yes	0	Classic
	**H110**	100+	1-15 mm	-	Yes	1	Classic
	**H111**	Multiple	Small	-	Yes	3	Classic
	**H112**	Multiple	Small	HGD	No	2	Attenuated
	**H113**	Carpeting	-	-	No	1	Classic
	**H114**	10+	-	-	No	3	Classic
	**H115**	Carpeting	-	-	Yes	0	Classic

- : not specified. Multiple: Unspecified number of polyps; still visible non-polyp regions. Carpeting: Innumerable polyps; little to no remaining non-polyp region. LGD: low-grade dysplasia. HGD: high-grade dysplasia. IM: intestinal metaplasia. *Duodenal phenotype described as Spigelman Stage 0-4.

Supplementary Table 2: Oligonucleotide primer sequences used for qRT-PCR gene expression analysis.

Gene	Amplicon size (bp)	Forward Primer (5' – 3')	Reverse Primer (5' – 3')
ACTB	211	CATCGAGCACGGCATCGTCA	TAGCACAGCCTGGATAGCAAC
HPRT	131	CCTGGCGTCGTGATTAGTGAT	AGACGTTCAGTCCTGTCCATAA
AQP5	139	TACGGTGTGGCACCGCTCAATG	AGTCAGTGGAGGCGAAGATGCA
ATP4A	265	ACAGATTGGTCAACGAGCCC	TGGCACACCTCAATGCTGAT
AXIN2	103	CTGGTGCAAAGACATAGCCA	AGTGTGAGGTCCACGGAAAC
CD44	151	CCAGAAGGAACAGTGGTTTGGC	ACTGTCCTCTGGGCTTGGTGTT
CD45	126	ACCACAAGTTTACTAACGCAAGT	TTTGAGGGGGATTCCAGGTAAT
CDKN1C	138	AGATCAGCGCCTGAGAAGTCGT	TCGGGGCTCTTTGGGCTCTAAA
CHIA	143	AAGGCTACACTGGAGAGAACAG	GGTAGGGAATCCAACGATGAGC
IL-1B	159	ATTTCTTGCTATTGACCGATGC	CCCAAGGAGACCACAGTTAGAG
IL-8	256	CTGAGCCCTGAACACCAGAG	CCTCTTTGGCCTCTTCCCAG
GIF	150	TGCCCCAGGTCACTTGTAGT	TGGTCTCGTTGAAGAGCAGC
LGR5	159	CCTATCGTCCAACCTCCTGTCG	GCACAGCACTGGTAAGCATAAGG
LIPF	127	TGACCTTCCAGCCACAATCGAC	TTTAGCCAGGCTGGGATTGGTG
MUC5AC	103	GGAACTGTGGGGACAGCTCTT	GTCACATTCCTCAGCGAGGTC
MUC6	122	GGACTGTGAGTGTCTGTGCGAT	GCGTGTTGTAGAAGCCGCAGTA
PGC	128	ACCTACTCCACCAATGGGCAG	TCACTCAAGCCGAACTCCTGGT
SP5	147	CTCGCTGCAGGCCTTTCT	TAGGGCACCTGCAGGAAGT
SOX9	116	TGCAGGAGGAGAAGAGAAGG	GTGGCCAGTTCACAGCTGC
TFF1	179	CCCAGTGTGCAAATAAGGGC	GCTCTGGGACTAATCACCGT
TFF2	228	GACAATGGATGCTGTTTCG	GTAATGGCAGTCTTCCACAGA
TNFRSF19	114	CAGGCATCTGAAAACTCGCCAC	GGTGCATTCTGCAGCCAGTCTT

Supplementary Table 3: Oligonucleotide primer sequences used for *APC* mRNA amplification.

***APC* Mutation Site**	**Patients**	**Amplicon size (bp)**	**Sequence (5' – 3')**
D1942	H72, H73	279	F: AACCTCCAACCAACAATCAGC
			R: GGGGGCTCAGTCTCTTTGATAG
A1585	H87	295	F: TGAAAACCAAGAGAAAGAGGCAG
			R: AACACAATACACCCGTGGCA

Figure S1

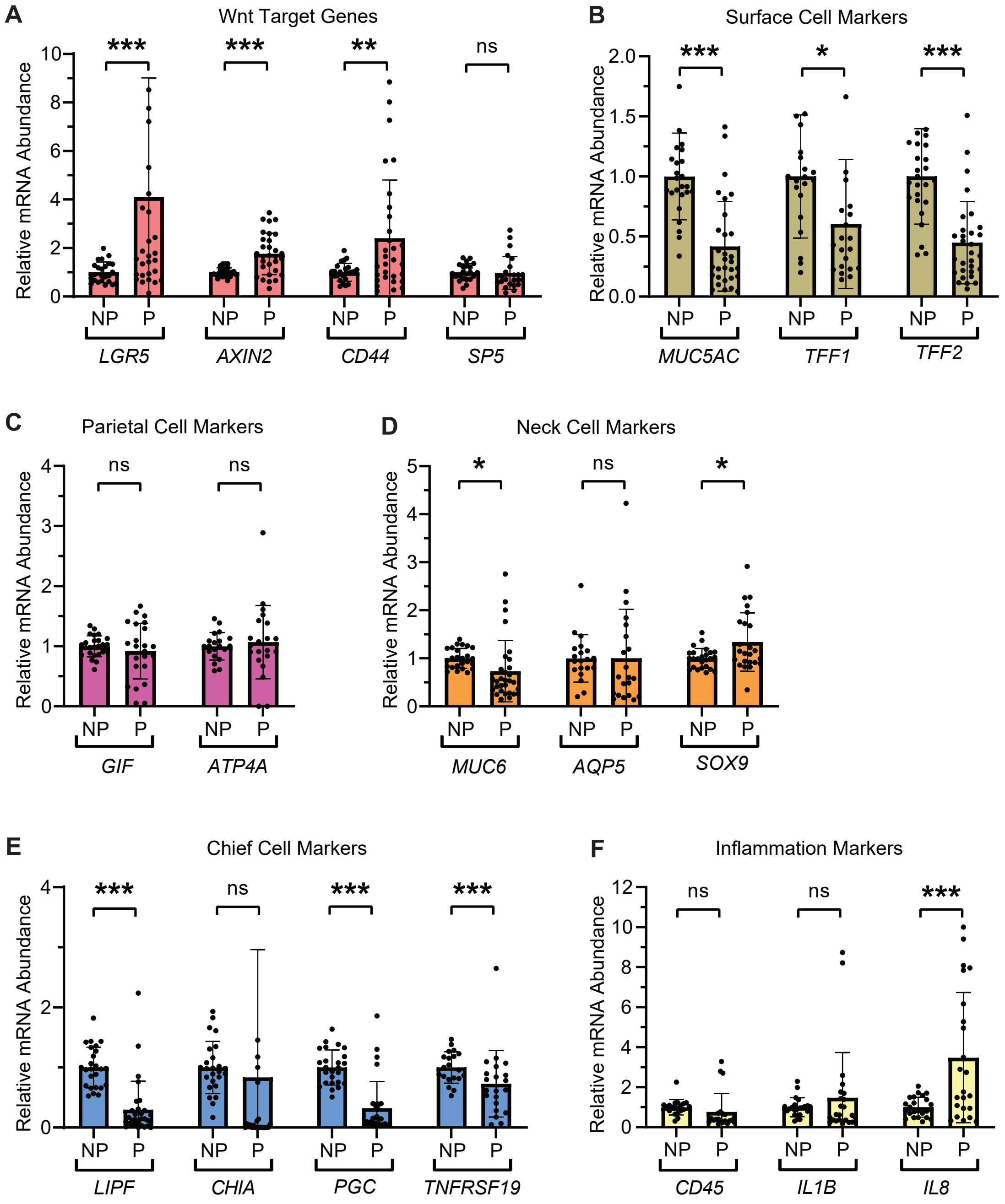

Figure S1: mRNA expression analysis of primary FGP and surrounding non-polyp biopsies from FAP patients. A-F) Relative mRNA abundance of (A) Wnt target genes, (B) surface mucous cell markers, (C) parietal cell markers, (D) mucous neck cell markers, (E) chief cell markers, and (F) immune cells/inflammatory markers was measured by qRT-PCR analysis of polyp (P) and non-polyp (NP) biopsies from 10 FAP patients. mRNA abundance was calculated as fold-change relative to the mean patient-matched non-polyp mRNA abundance and normalized within each patient data set, with *HPRT* used for a reference. Data are shown as mean ± SD ($n_{non\text{-}polyp}$ = 20-25 biopsies, n_{polyp} = 21-30 biopsies; *p<0.05, **p<0.005, ***p<0.001 by unpaired parametric *t* test).

Figure S2

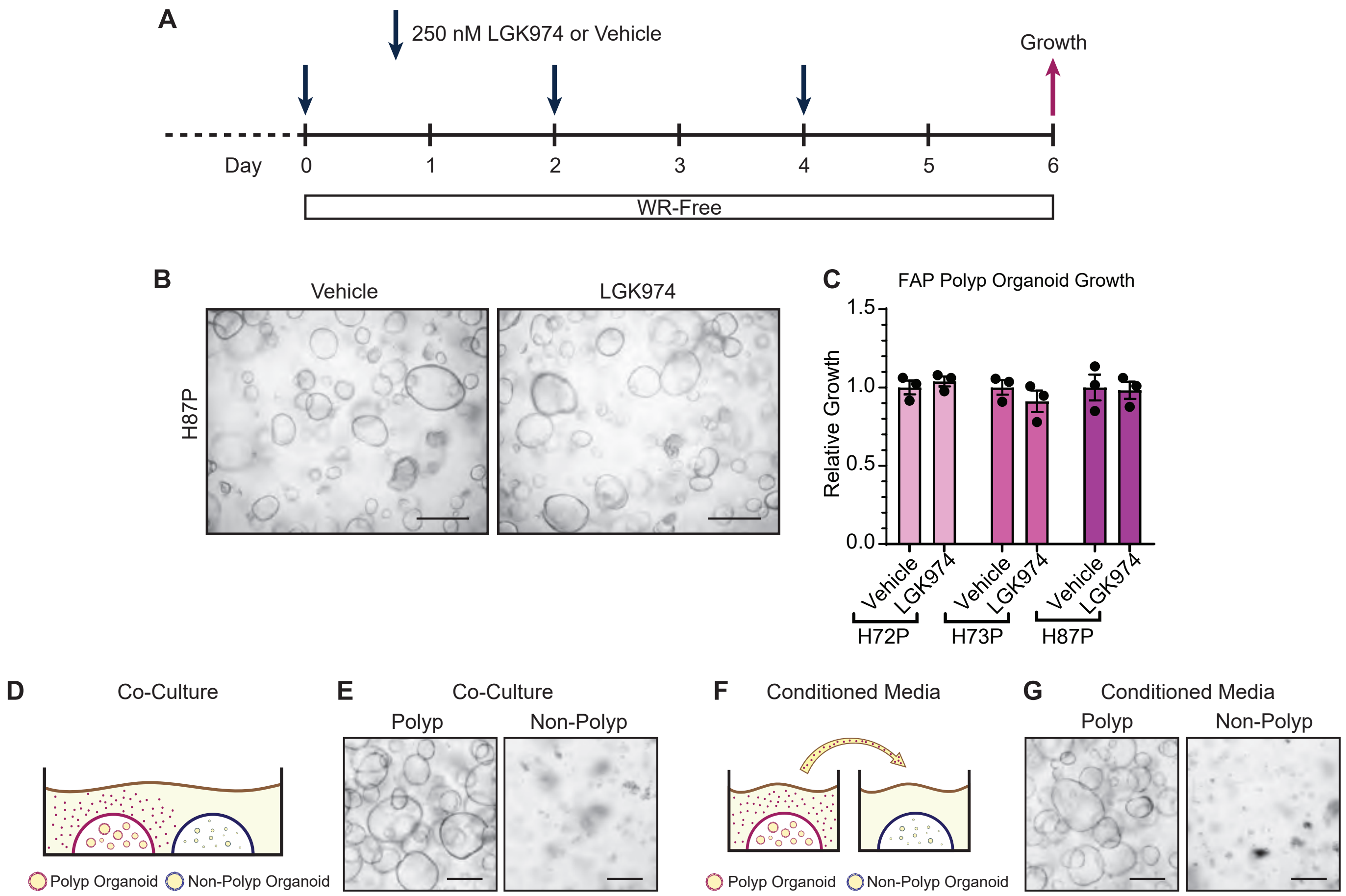

Figure S2: High Wnt FAP organoids are not sustained through secreted ligands. A) High Wnt polyp organoids demonstrating long-term Wnt independent growth in WR-Free media were treated with the porcupine inhibitor LGK974 (250 nM) or DMSO (Vehicle). Growth was measured through ATP-dependent luminescence on day 6. B) Representative images of H87P organoids grown in WR-Free media on day 6 following treatment with Vehicle or LGK974 (size bars = 200 μm). C) Relative growth of Vehicle- or LGK974-treated H72P, H73P, or H87P organoids (n = 3 triplicate wells). D) Schematic of co-culture experiment of non-polyp organoids within the same well as polyp organoids from High Wnt lines that had been growing for several passages in WR-Free media. E) Representative images of H87P and H87NP organoids after six days of co-culture in WR-Free media (size bars = 100 μm). F) Schematic demonstrating treatment of non-polyp organoids with conditioned media derived from High Wnt polyp organoids grown six days in WR-Free media. G) Representative images of H87P and H87NP organoids following growth for six days in High Wnt polyp conditioned media (size bars = 100 μm).

Figure S3

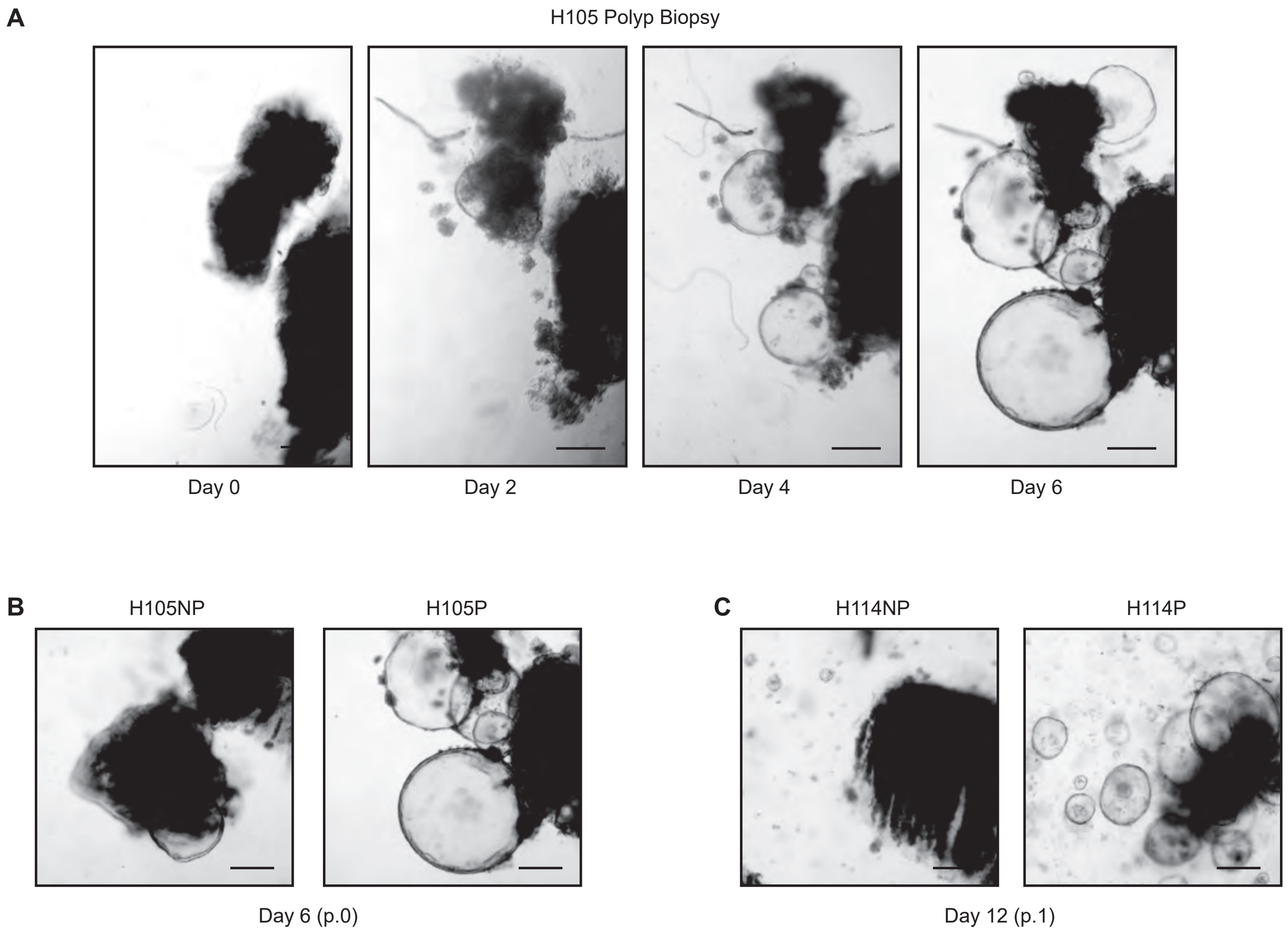

Figure S3: Establishment of human FAP gastric organoids from biopsies. FAP patient biopsies were minced, embedded in Matrigel and cultured in 60% WR media. A) Representative images of epithelial outgrowth from primary FGP biopsy from patient H105 embedded in Matrigel (size bars = 100 μm). B) Representative images of epithelial outgrowth from non-polyp (H105NP) and polyp (H105P) biopsies six days after initial embedding in Matrigel (size bars = 100 μm). C) Representative images of epithelial outgrowth from non-polyp (H114NP) and polyp (H114P) biopsies six days after the first passage (12 days from initial seeding) (size bars = 100 μm).

Figure S4

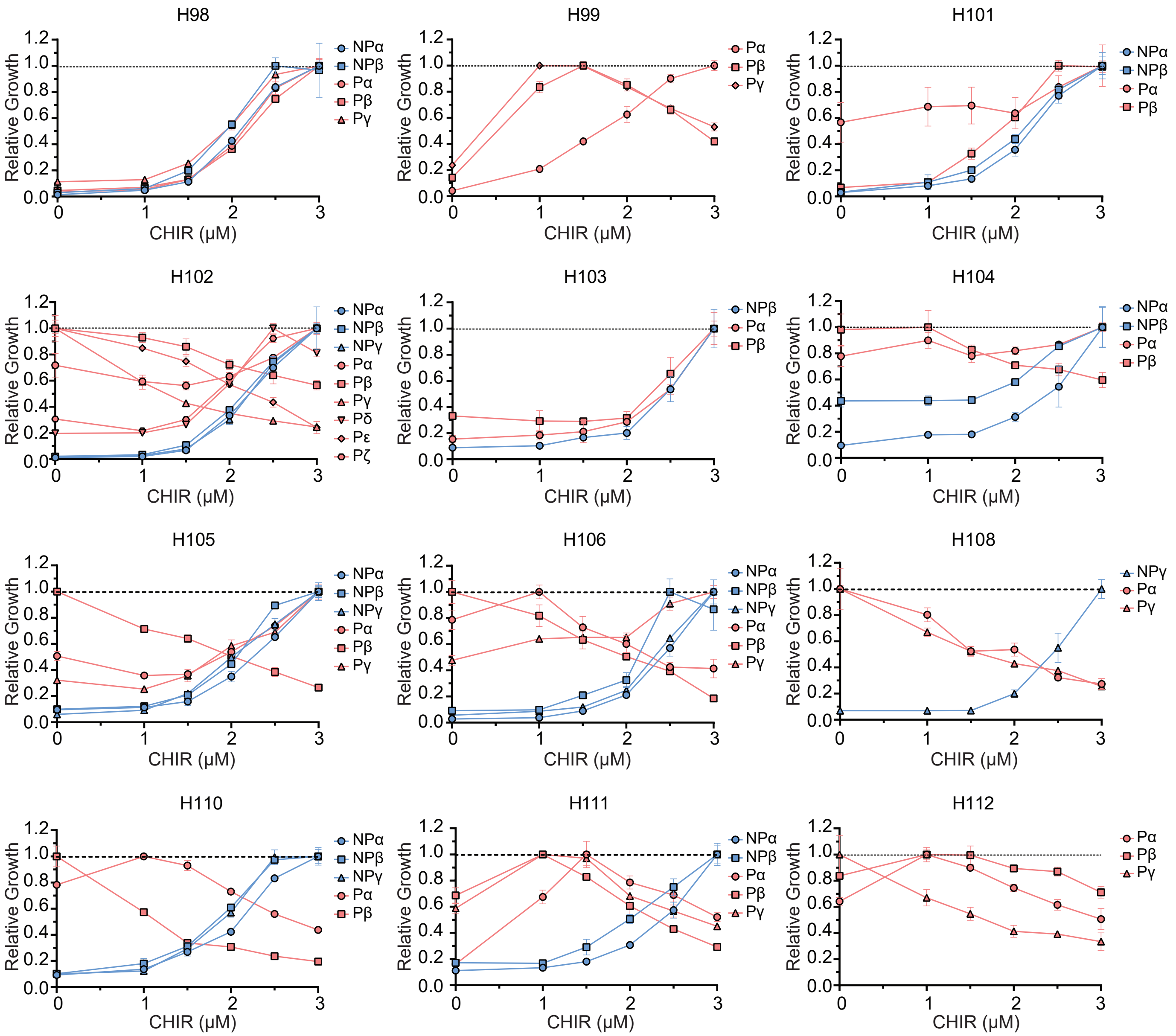

Figure S4: Polyp-derived FAP organoids exhibit significant intra-patient variability in Wnt dependence. Relative growth of established polyp and non-polyp organoid lines from respective FAP patients measured on day 5 after growth in CHIR99021 (experimental design shown in Figure 3B). Growth rates were normalized to the maximum observed growth rate observed for each individual line. Data are presented as mean ± SD of triplicate wells for each line. Blue lines represent non-polyp organoids. Red lines represent polyp organoids. These data complement the summary data shown in Figure 3.

Figure S5

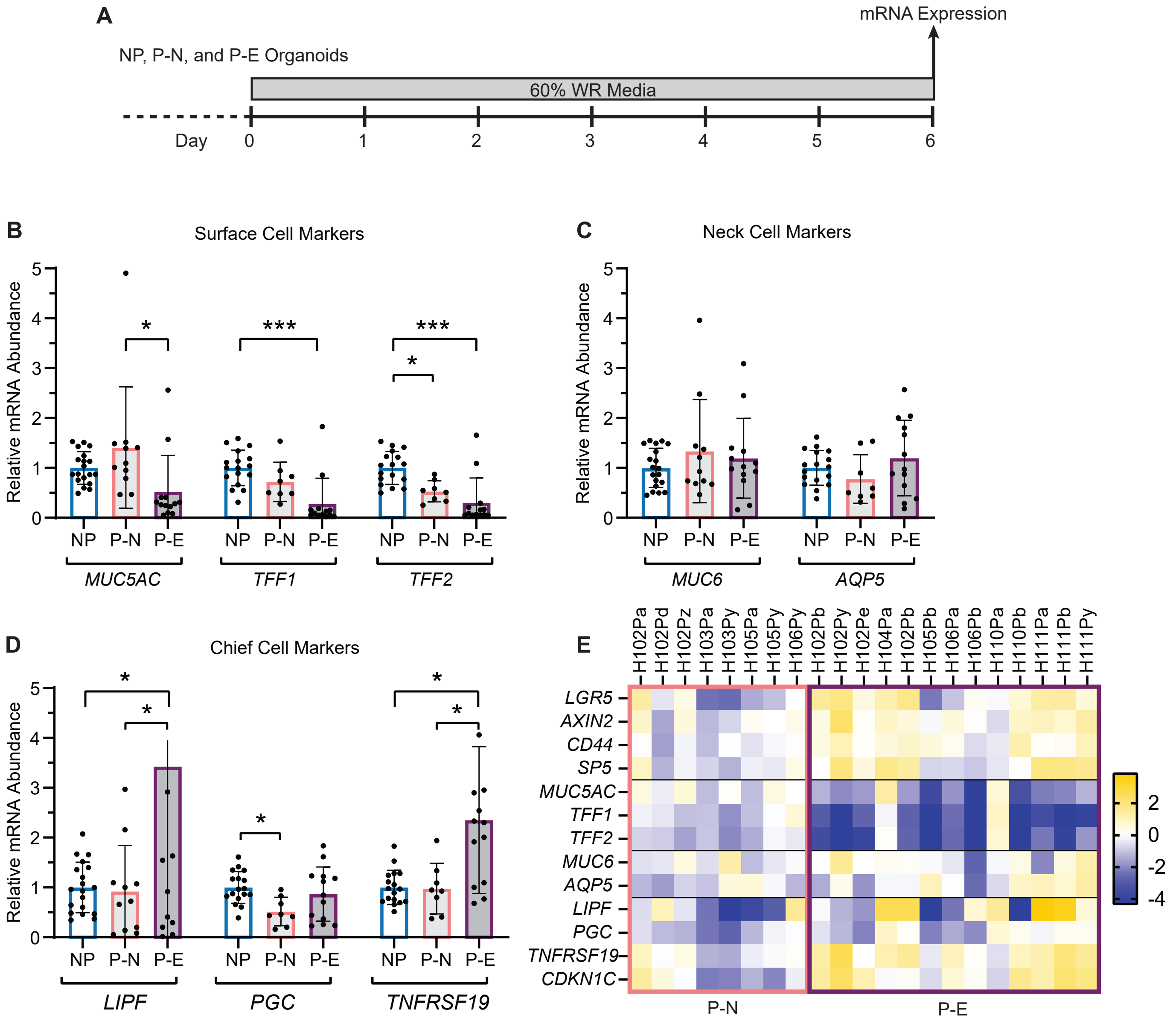

Figure S5: Gene expression in FAP organoids. A) RNA was extracted from established polyp and non-polyp FAP organoids following six days growth in 60% WR media. Organoids were categorized as non-polyp (NP, n = 19); normal-like polyp (P-N, n = 11), and enhanced polyp (P-E, n = 13) as described in the text and Figure 3. B-D) Relative mRNA abundance of (B) surface, (C) neck, and (D) chief cell markers. Data are shown as mean ± SD fold change relative to patient-matched non-polyp organoids and normalized across all lines, with *HPRT* used as a reference. (*p<0.05, ***p<0.001 by one-way ANOVA with Tukey's multiple-comparison test). E) Heatmap of mRNA expression for individual polyp organoid lines. Data are presented as Log(2) fold-change expression in polyp organoids relative to average of non-polyp organoids from the same patient. Lines outlined in pink (left box) are normal-like polyps (P-N). Lines outlined in purple (right box) are enhanced polyps (P-E).

Figure S6

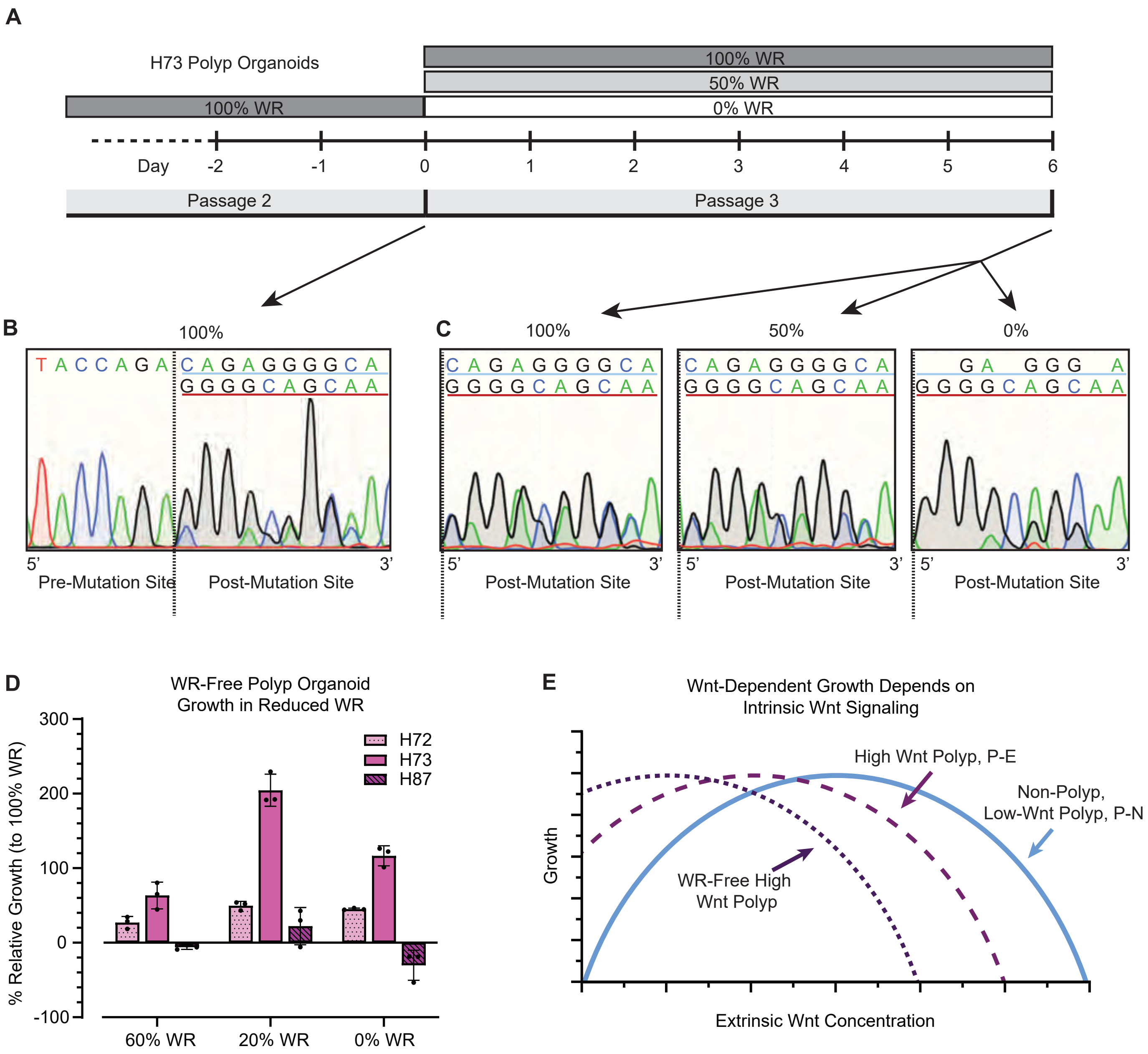

Figure S6: WR-Free media selects for organoids with transcriptional loss of wildtype *APC* expression. A) H73 polyp organoids (passage 2) were grown from initial *in vitro* establishment in 100% WR, then transitioned to 100%, 50%, or 0% WR media during passage 3. mRNA was harvested at the conclusion of passage 2, or on day 6 of passage 3. B) Chromatogram of sequenced *APC* cDNA harvested from H73 polyp organoids at the conclusion of passage 2. The blue underlined sequence aligned with the normal sequence. The red underline sequence aligned with the mutant sequence. C) Chromatogram of sequenced *APC* cDNA harvested from H73 polyp organoids following growth for passage 3 in 100%, 50%, or 0% WR. The sequence shown includes the 10 base pairs immediately downstream of the familial mutation site (see Figure 4D). The blue underlined sequence aligned with the normal sequence. The red underlined sequence aligned with the mutant sequence. D) H72, H73, and H87 polyp organoids were grown for 3+ passages in WR-Free media to establish long-term Wnt independent growth, then grown for 12 days in 100%, 60%, 20%, or 0% WR. Values are polyp organoid growth at day 12 in 60%, 20%, or 0% WR relative to growth in 100% WR, following the same procedure outlined in Figure 2F. E) Schematic of relationship between intrinsic Wnt characteristics and extrinsic Wnt ligand in driving growth in accordance with the 'just-right' hypothesis.

Figure S7

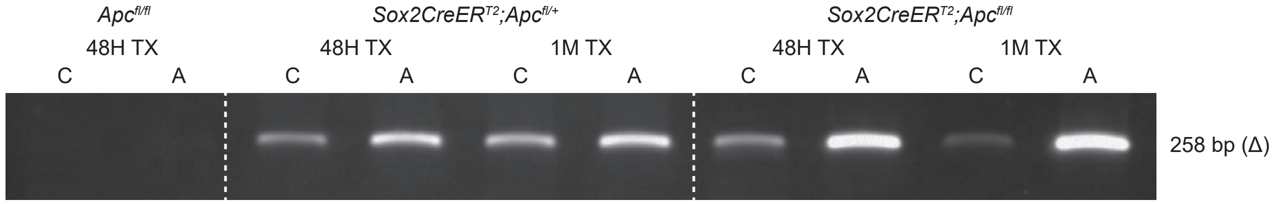

Figure S7: Heterozygous loss of *Apc* is maintained long-term within the corpus epithelium. Agarose gel showing PCR products for the recombined *Apc*^{fl} allele (258 bp) at 48 hours (48H) and one month (1M) post-tamoxifen. DNA was isolated from full-thickness corpus (C) or antrum (A) of *Apc*$^{fl/fl}$ (control), *Sox2CreER*^{T2}*; Apc*$^{fl/+}$ (heterozygous), or *Sox2CreER*^{T2}*; Apc*$^{fl/fl}$ (homozygous) mice.

Figure S8

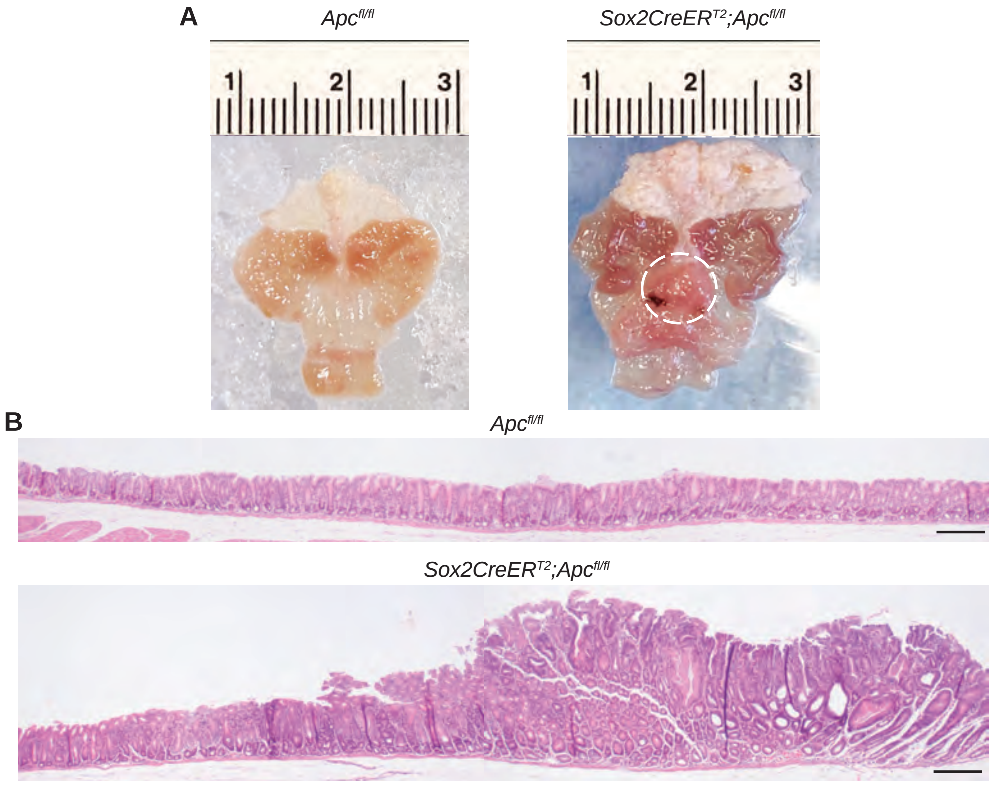

Figure S8: Antral polyps develop in FAP mouse model with homozygous *Apc* mutation. A) Gross images of control (*Apc*$^{fl/fl}$) and homozygous (*Sox2CreERT2;Apc*$^{fl/fl}$) stomachs one month after *Apc* deletion was induced by tamoxifen treatment (see Figure 5A). Circled area: antral polyp. B) H&E stained paraffin sections from control and homozygous Apc mutant antrum (size bars = 200 µm).
